# Intrathymic differentiation of natural antibody-producing plasma cells in human neonates

**DOI:** 10.1038/s41467-021-26069-2

**Published:** 2021-10-01

**Authors:** Hector Cordero, Rodney G. King, Pranay Dogra, Chloe Dufeu, Sarah B. See, Alexander M. Chong, Anne-Catrin Uhlemann, Siu-Hong Ho, David M. Kalfa, Emile A. Bacha, John F. Kearney, Emmanuel Zorn

**Affiliations:** 1 Columbia Center for Translational Immunology, Columbia University Medical Center, New York, NY 10032 USA; 2 Department of Microbiology, University of Alabama at Birmingham, Birmingham, AL 35294 USA; 3 Department of Microbiology and Immunology, Columbia University Medical Center, New York, NY 10032 USA; 4 Division of Infectious Diseases in the College of Physicians and Surgeons, Columbia University Medical Center, New York, NY 10032 USA; 5 Division of Cardiac, Thoracic and Vascular Surgery, Columbia University Medical Center, New York, NY 10032 USA

**Keywords:** Lymphopoiesis, Antimicrobial responses, B cells

## Abstract

The thymus is a central lymphoid organ primarily responsible for the development of T cells. A small proportion of B cells, however, also reside in the thymus to assist negative selection of self-reactive T cells. Here we show that the thymus of human neonates contains a consistent contingent of CD138^+^ plasma cells, producing all classes and subclasses of immunoglobulins with the exception of IgD. These antibody-secreting cells are part of a larger subset of B cells that share the expression of signature genes defining mouse B1 cells, yet lack the expression of complement receptors CD21 and CD35. Data from single-cell transcriptomic, clonal correspondence and in vitro differentiation assays support the notion of intrathymic CD138^+^ plasma cell differentiation, alongside other B cell subsets with distinctive molecular phenotypes. Lastly, neonatal thymic plasma cells also include clones reactive to commensal and pathogenic bacteria that commonly infect children born with antibody deficiency. Thus, our findings point to the thymus as a source of innate humoral immunity in human neonates.

## Abstract (web summary)

The thymus is known as the organ of T lymphocyte development. Here authors show that terminal B cell differentiation also takes place in the thymus of human neonates, leading to antibody production against commensal and pathogenic bacteria.

## Introduction

The thymus is primarily responsible for the development, maturation, and selection of T cells. As recognized more than 30 years ago, this lymphoid organ also houses a functional population of CD19^+^ B cells^1^. In both mice and humans, B cells account for ~0.5% of the thymic cellularity^1,2^. While still under investigation, thymic B cells are now known to play an important role in the induction of the central tolerance through the negative selection of self-reactive T cells^2–5^. This function is in accordance with their location in the thymic medulla and the expression of key co-stimulatory molecules such as CD80, CD86 as well as high levels of MHC class II molecules^2,3,5,6^. The autoimmune regulator (AIRE) expressed in medullary thymic epithelial cells (mTECs) and involved in the negative selective of peripheral antigen-specific T cells was also reported in murine thymic B cells^5^, supporting a similar role as antigen presenting cells. Additionally, murine thymic B cells were involved in the differentiation of thymocytes into FOXP3-expressing regulatory T cells (Tregs), which also takes place in the medulla^7,8^. Lastly, the presence of class-swtiched antibody-producing thymic B cells was also reported and involved in mechanisms of central tolerance^9^. Collectively, these animal studies have begun uncovering the important role of thymic B cells in immune regulatory mechanisms. In humans, our understanding of these cells is far less advanced. The disctinction between the two species is particularly relevant because human and mouse thymuses differ structurally^10,11^. Moreover, the aging process of the thymus in mice maintained in conventional laboratory conditions does not reflect physiological conditions^10^.

We recently reported a longitudinal study of the distribution of B cells in human thymuses using specimens from donors aged 5 days–71 years. Our investigation showed that while B cells were mostly distributed in the thymic medulla in newborns and infants, there was also a progressive accumulation of these cells in the perivascular space (PVS), starting during the first year of life^6^. The PVS constitutes a third thymic compartment that expands with age concomitantly with the involution of the cortex^12,13^. Furthermore, our studies demonstrated that B cells accumulating with age in the thymic PVS included antibody-secreting cells (ASC) specific to common viruses and vaccine antigens^6^. The specificity of these ASC to immunizing antigens as well as the kinetics of their accumulation strongly suggested that they resulted from peripheral immune response. These findings revealed an unrecognized role of the thymic PVS as a functional lifetime niche for viral-specific plasma cells (PCs).

Here we examine the thymic B cell heterogeneity of human neonates and uncover a consistent subset of fully differentiated CD138^+^ plasma cells. We reveal that these plasma cells differentiate intrathymically and secrete immunoglobulins with a reactivity profile characteristic of natural antibodies. Overall, this study establishes the thymus as a site of natural antibody production in human neonates.

## Results

### Neonatal thymus houses ASCs comprised within CD21^−^CD35^−^ B cell subset

**Fig. 1:**
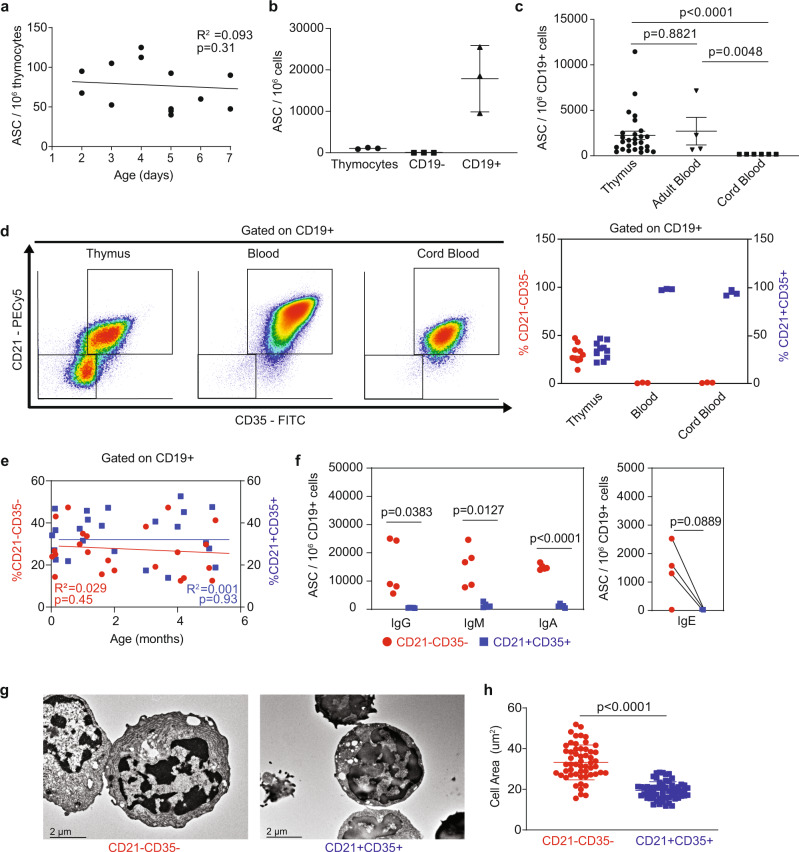
The human neonatal thymus contains antibody-secreting cells. **a** Number of IgG-ASC during the first week of life measured by ELISpot (*n* = 13). Coefficient of determination (R^2^) test. **b** Number of IgG-ASC per million of total, CD19^−^ and CD19^+^ thymocytes measured by ELISpot (*n* = 3). Bars are defined as mean values ± standard desviation (SD). **c** Number of IgG-ASC per CD19^+^ B cells in the thymus (*n* = 27), adult blood (*n* = 4), and cord blood (*n* = 6). Bars are defined as mean values ± standard error of the mean (SEM). Two-sided Mann–Whitney was performed. **d** Expression of CD21 and CD35 by thymus, adult blood, and cord blood CD19^+^ B cells measured by flow cytometry. Representative profiles are shown in left panels. Mean percentages are shown in the right panel. Error bars are expressed as mean ± SD (*n* = 5). **e** Percentage of thymic CD21^-^CD35^-^ B cell subset (red dots) and CD21^+^CD35^+^ B cell subset (blue dots) as a function of age during the first six months of life (*n* = 21). Coefficient of determination (R^2^) test was calculated for each thymic B cell subset. **f** Number of IgG, IgM, IgA, and IgE-ASCs within thymic CD21^−^CD35^−^ and CD21^+^CD35^+^ B cells (*n* = 5). **g** Transmission electron microscopy (TEM) representative microphotographs of thymic CD21^−^CD35^−^B cell subset (left) and CD21^+^CD35^+^ B cell subset (right). This experiment was repeated independently three times with similar results. **h** Cell area of thymic CD21^−^CD35^−^ and CD21^+^CD35^+^ B cells measured by imaging analysis using ImageJ (*n* = 50). Error bars represent standard desvitation (SD). Two-sided *t*-test was performed.

In previous studies, we showed that the human thymus provides a niche supporting antibody-producing plasma cells^6^. While the number of thymic ASC increases with age, starting during the first year of life, their presence at birth had not been fully evaluated. Here, we characterized ASCs in thymic specimens (*n* = 12) obtained up to 7 days after birth. This restricted time window ensured that we examined ASCs that had been previously differentiated in utero. Cells secreting IgG ex vivo without stimulation represented ~1 in 10^4^ thymocytes and this frequency was consistent within the first week of life (Fig. 1a, Supplementary Fig. 1a). The spontaneous secretion of immunoglobulins without the need for in vitro stimulation is a hallmark of terminally differentiated plasma cells such as long-lived plasma cells (LLPC) residing in the bone marrow niche. However, while bone marrow LLPC lose the expression of CD19^14^, thymic ASC were almost exclusively contained in the CD19^+^ fraction (Fig. 1b, Supplementary Fig. 1b). The frequency of thymic CD19^+^ cells is between 0.5 and 5% of total thymocytes in neonates, for a total of 1–2 × 10^8^ B cells in an average-sized thymus weighing 12 g. The overall frequency of thymic ASC was equivalent to that of CD19^+^ ASC in adult blood. In contrast, no circulating ASC were detected in cord blood (Fig. 1c, Supplementary Fig. 1c), suggesting that thymic PC developed in situ and did not recirculate during perinatal stages.

The differential expression of CD21 by peripheral blood B cells has been reported to define functionally distinct B cell subsets following influenza vaccination^15^. Thymic B cells are heterogeneous, with an important subset previously reported to lack expression of both complement receptors CD21 and CD35^1^. We hypothesized that these two complement receptors could potentially be lost or internalized after B cell activation in the thymus. Using these two markers, thymic CD19^+^ B cells could be subdivided into CD21^−^CD35^−^ and CD21^+^CD35^+^ populations (Fig. 1d). CD21^−^CD35^−^ B cells were not present in adult or cord blood. Both subsets were consistently detected in thymus specimens collected within the first 6 months after birth (*n* = 21; Fig. 1e). Based on these findings, we selected 5 representative thymus specimens ranging in age between 1 day and 4 months for in-depth analysis. CD21^−^CD35^−^ and CD21^+^CD35^+^ Thymic B CD19^+^ B cells were sorted as described in Supplementary Fig. 1d and used in ELISpot assay to detect immunoglobulin secretion. All ASC were included in the CD21^−^CD35^−^ subset and spontaneously secreted IgG, IgM, IgA, and in some cases IgE (3 out of 5) (Fig. 1f, Supplementary Fig. 1e, Supplementary Data 1). While we had previously reported on the presence of IgG, IgM, and IgA-secreting cells in the human thymus^6^, IgE-secreting cells had not been detected. The diversity of immunoglobulin classes produced by neonatal thymic ASC points to active class-switch recombination in utero.

Early studies using transmission electron microscopy (TEM) and immunohistochemistry described subsets of large “asteroid in shape” B cells together with smaller round B cells in the fetal and adult thymic medulla, highlighting the heterogeneity of this population^16,17^. We also used transmission electron microscopy to examine the cellular structure of CD21^−^CD35^−^ and CD21^+^CD35^+^ B cells. Overall, CD21^−^CD35^−^ B cells were morphologically different from CD21^+^CD35^+^ B cells, displaying intracellular organelles and size consistent with activated B cell blasts as well as typical plasma cells with enlarged rough endoplasmic reticulum and cart-wheel-shaped nuclear patterns (Fig. 1g, h, Supplementary Fig. 1f). This subset of blastic B cells likely corresponds to the asteroid B cells mentioned previously.

### Neonatal thymic B cell subsets display a unique innate-like B cell gene signature

**Fig. 2:**
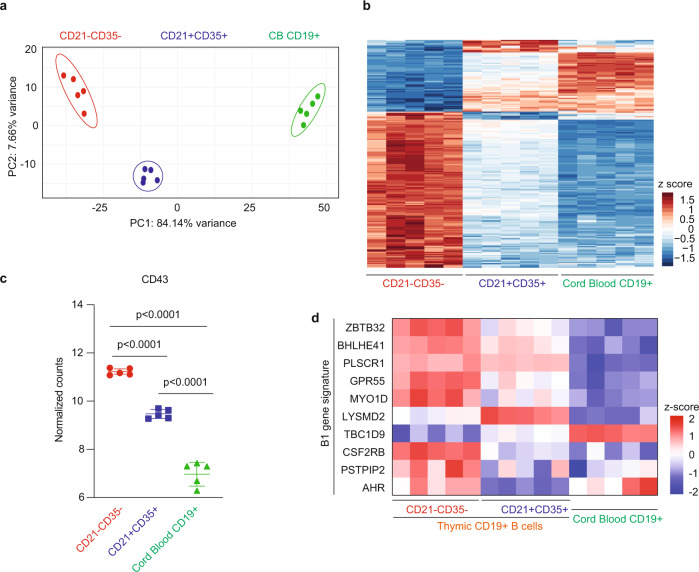
Comparative transcriptome analysis of thymic B cell subsets and cord blood B cells. **a** Principal component analysis (PCA) clustering of thymic CD21^−^CD35^−^ (red dots), CD21^+^CD35^+^ (blue dots) and cord blood (green dots) B cells using top 500 most variable genes (*n* = 5). **b** Heatmap of thymic CD19^+^CD21^−^CD35^−^ and CD19^+^CD21^+^CD35^+^ B cell subsets as well as cord blood CD19^+^ B cells after DESeq2 analysis pipeline. Data are expressed as normalized row z-score of log2 gene expression values (*n* = 5). Two-sided Wald test with Benjamini–Hochberg false discovery ration (FDR) adjustment. **c** Normalized counts of *CD43* gene expression in thymic CD19^+^CD21^−^CD35^−^, CD19^+^CD21^+^CD35^+^, and cord blood CD19^+^ B cell subsets after DESeq2 analysis pipeline. Error bars are expressed as mean ± SD (*n* = 5); two-sided *t*-test. **d** Heatmap representation of expression of genes included in the canonical mouse B1 cell signature in human thymic CD21^−^CD35^−^, CD21^+^CD35^+^ CD19^+^ B cell subsets as well as human cord blood CD19^+^ cells. Data are expressed as normalized row z-score of log2 gene expression values (*n* = 5). Two-sided Wald test with Benjamini–Hochberg false discovery ration (FDR) adjustment.

We next used RNA-seq to further characterize neonatal thymic B cell subsets and cord blood B cells as a comparison (Supplementary Data 1). Volcano plot showed a total of 5639 DE genes (2976 upregulated, 2663 downregulated) between thymic B cell subsets when compared to cord blood B cells (Supplementary Fig. 2a). Principal component analysis (PCA) segregated all three populations of B cells (Fig. 2a). In addition, unsupervised clustering revealed marked differences between the gene expression signature of thymic CD21^−^CD35^−^ B cells when compared to thymic and cord blood CD21^+^CD35^+^ B cells (Fig. 2b). Among the differentially expressed genes, *SPN* (sialophorin) encoding *CD43* a marker associated with B1 cells in mice, was upregulated in both thymic B cell subsets when compared to cord blood B cells (Fig. 2c). We then focused on a select set of genes established as the minimal signature of B1 and B2 B cells in mice^18,19^. More than 50% of the genes defining the canonical B1 signature in mice and detected in our transcriptomics analysis, were upregulated in both CD21^−^CD35^−^ and CD21^+^CD35^+^ thymic B cell subsets when compared to cord blood B cells, including *ZBTB32*, *BHLHE41*, *PLSCR1*, *GPR55*, and *MYO1D* (Fig. 2d). Although these individual genes are not exclusively restricted to B1 B cells. In contrast, only one gene included in the B2 signature was differentially regulated between these B cell populations (Supplementary Table 1). Of note, we could not detect the expression of *AIRE* in thymic CD21^−^CD35^−^ or CD21^+^CD35^+^ B cells (Supplementary Data 1) as was previously reported for mouse thymic B cells^2,5,20^, suggesting a divergence between the two species.

### The neonatal thymic CD21^−^CD35^−^ B cell subset is highly heterogenous

**Fig. 3:**
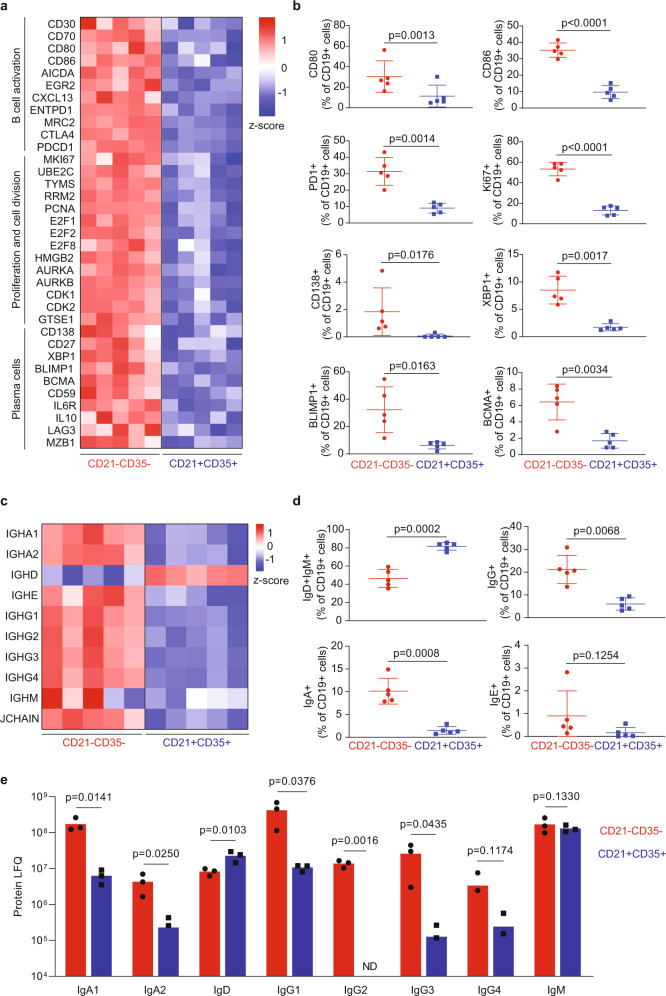
Transcriptome profiling of thymic CD21^−^CD35^−^ and CD21^+^CD35^+^ B cell subsets in human neonates. **a** Heatmap representation of selected genes differentially expressed between thymic CD21^−^CD35^−^ B cell subset (left side) and CD21^+^CD35^+^ B cell subset (right side) involved in B cell activation, class-switch recombination, proliferation, and plasma cell differentiation. Gene values were expressed as normalized row z-score of log2 gene expression values (*n* = 5). Two-sided Wald test with Benjamini–Hochberg false discovery ration (FDR) adjustment. **b** Frequency of CD80^+^, CD86^+^, PD1^+^, KI67^+^, CD138^+^, XBP1^+^, BLIMP1^+^, and BCMA^+^ cells within CD19^+^CD21^−^CD35^−^ and CD19^+^CD21^+^CD35^+^ subsets in the thymus of neonates and infants aged 1 day–4 months, measured by flow cytometry (*n* = 5). Bars are defined as mean values ± SD. Two-sided *t*-test was performed. **c** Heatmap representation of transcripts of immunoglobulin genes in CD19^+^CD21^−^CD35^−^ and CD19^+^CD21^+^CD35^+^ thymic B cells. Gene values were expressed as normalized row z-score of log2 gene expression values. Two-sided Wald test with Benjamini–Hochberg false discovery ration (FDR) adjustment. **d** Frequency of IgD^+^IgM^+^, IgG^+^, IgA^+^, and IgE^+^ cells within CD19^+^CD21^−^CD35^−^ and CD19^+^CD21^+^CD35^+^ subsets in the thymus of neonates and infants aged 1 day–4 months, measured by flow cytometry (*n* = 5). Bars are defined as mean values ± SD. Two-sided *t*-test was performed. **e** Protein levels of different classes and subclasses of immunoglobulins in CD19^+^CD21^−^CD35^−^ and CD19^+^CD21^+^CD35^+^ thymic B cells measured by iST proteomics and expressed as protein label-free quantification (LFQ, *n* = 3; ND = no detected). Bar charts are defined as mean values, dots are individual values. Two-sided *t*-test was performed.

A total of 1800 genes were differentially expressed between thymic CD21^+^CD35^+^ and CD21^−^CD35^−^ B cell with 1120 upregulated and 680 downregulated genes in the thymic CD21^−^CD35^−^ B cell subset (Supplementary Fig. 2b, Supplementary Data 2). Geneset enrichment analysis (GSEA) identified BCR signaling, B cell activation, cell cycle, and defense responses to bacteria among the most upregulated pathways in CD21^−^CD35^−^ B cells (Supplementary Table 2, Supplementary Fig. 3a). Several key genes associated with T cell interaction, such as *CD80* and *CD86*, were upregulated in thymic CD21^−^CD35^−^ B cells (Fig. 3a), supporting a role previously attributed to medullary B cells in T-cell-negative selection^2,5^. Other activation-induced genes expressed in CD21^−^CD35^−^ B cells included *CD30*, *CD70*, *CTLA-4* as well as *PDCD1*, coding for programmed cell death protein 1 (PD1). Moreover, the expression of *AICDA* further confirmed intrathymic class-switch recombination (CSR) previously reported in mice^9^. Thymic CD21^−^CD35^−^ B cells in neonates also displayed upregulation of genes involved in cell division and proliferation such as *MKI67*, *UBE2C*, *THYMS*, *RRM2*, and *PCNA* (Fig. 3a). Moreover, several genes associated with B cell differentiation into plasmablasts and PC were upregulated in CD21^−^CD35^−^ B cells when compared to CD21^+^CD35^+^ cells. These include *CD138*, *XBP1*, *MZB1*, *IL6R*, *BLIMP1*, and *BCMA* (Fig. 3a). Differential expression of several of these key markers in CD21^−^CD35^−^ B cells was confirmed by high-dimensional flow cytometry (Fig. 3b, Supplementary Fig. 3b). In particular, 50–60% of the CD21^−^CD35^−^ B cells were positive for MKI67, indicating active proliferation of these cells.

In accordance with the upregulation of *AICDA* and class-switch recombination, the transcriptome profile of CD21^−^CD35^−^ thymic B cells revealed higher expression of genes encoding all classes and subclasses of immunoglubulins with the exception of *IGHM* and *IGHD* when compared to CD21^+^CD35^+^ B cells (Fig. 3c). A majority of immunoglobulin heavy (75%), kappa (83%), and lambda (98%) light chains variable region genes were upregulated in CD21^−^CD35^−^ cells with the notable exception of *IGHV5.78* that had previously been identified as a pseudogene (Supplementary Fig. 4a). The gene coding for the J chain was also upregulated in the double complement receptor negative B cell subset. Differential membrane expression of these immunoglobulins in CD21^−^CD35^−^ and CD21^+^CD35^+^ cells was verified by flow cytometry (Fig. 3d, Supplementary Fig. 4b). To further confirm these findings, we carried out an in-stage tip proteomics analysis of sorted CD19^+^CD21^−^CD35^−^ and CD19^+^CD21^+^CD35^+^ thymic B cells. The results verified overexpression of IgA1, IgA2, IgG1-3 but not IgM in CD21^−^CD35^−^ B cells (Fig. 3e). In addition, the cells that had undergone CSR showed lower levels of IgD. In contrast, CD21^+^CD35^+^ B cells displayed a late transitional/naïve phenotype characterized by the expression of CD19, CD21, IgD but the lack of CD27 and CD38 (Fig. 3c, Supplementary Fig. 3c).

### Intrathymic differentiation of B cells into plasma cells in human neonates

**Fig. 4:**
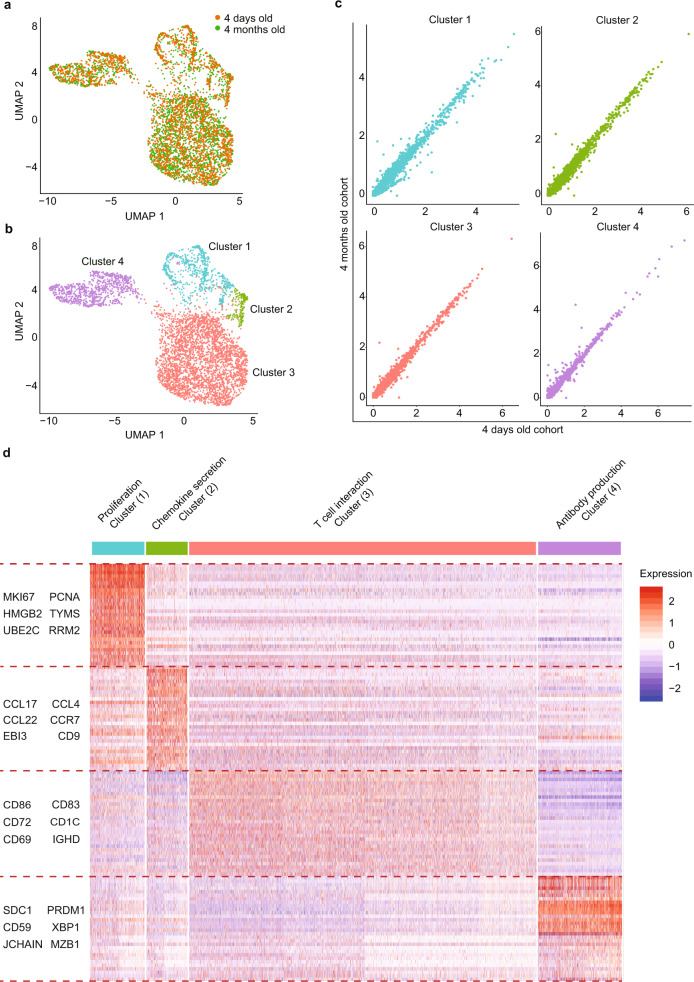
Single-cell RNA-sequencing analysis of thymic B cell in human neonates. **a** UMAP embeddings of integrated scRNA-seq data from thymic CD19^+^CD21^−^CD35^−^ B cells of representative donors aged 4 days (orange) and 4 months (green). **b** UMAP embeddings of scRNA-seq data from CD19^+^CD21^−^CD35^−^ thymic B cells of representative donors aged 4 days and 4 months, showing four different expression clusters. **c** Average of expression levels for each gene (dot) comparing 4-days-old specimens (*n* = 3) and 4-month-old specimens (*n* = 3) in each cell cluster. Values are expressed as natural log of average gene expression +1. **d** Heatmap representation with highlighted genes expressed in different clusters of thymic CD19^+^CD21^−^CD35^−^ B cells based on single-cell RNA-sequencing analysis of thymic B cells. Results are expressed as normalized row z-score of log values. Two-sided MAST (Model-based Analysis of Single-cell Transcriptomics) test with Bonferroni correction across gene dataset.

Bulk RNA-sequencing findings suggested a high heterogeneity within CD21^−^CD35^−^ thymic B cell subset. We further characterized these cells using single-cell RNA sequencing (scRNA-seq) in three 4-day-old and three 4-month-old specimens. An initial resolution of 0.1 was used for clustering and intercluster DE gene annotation. However, this analysis gave us three clusters with similar expression profile (Supplementary Fig. 5a). The integrated single-cell dataset was projected in a two-dimensional space using Uniform Manifold Approximation and Projection (UMAP). This approach revealed four different B cell clusters that were comparable in terms of cell distribution and gene expression between 4-day-old and 4-month-old thymus specimens (Fig. 4a–c, Supplementary Fig. 5b, 6). The first cluster corresponded to cells expressing genes involved in cell cycle progression and proliferation such as *MKI67*, *UBEC2B*, *TYMS*, *HMGB2*, and *PCNA* (Fig. 4d, Supplementary Fig. 7a). These genes matched those reported in Fig. 3a and corresponded to actively dividing cells. The second cluster, smaller in size, included B cells expressing *CCL22* and *CCL17*, two chemokines involved in the chemotaxis of CCR4-expressing Th2 and Tregs^21,22^. B cells in this cluster also expressed the tetraspanin *CD9*, a marker of IL-10-producing regulatory B cells^23,24^ and *EBI3*, expressed in regulatory plasma cells^25^ and involved in CD4 T cell regulation^26^ and tolerance^27^ (Fig. 4d, Supplementary Fig. 7a). The third and largest cluster was characterized by expression of *CD86*, *CD83*, *CD72*, and *CD1c*, indicating that B cells in this cluster are well equipped to interact with T cells^28–30^. This cluster also included B cells expressing markers associated with a mature naïve phenotype, such as *IgD*^31^. Lastly, the fourth cluster was characterized by genes expressed in plasmablasts and plasma cells, including *SDC1* (*CD138*), *MZB1*, *XBP1*, *PRDM1*, and *JCHAIN* (Fig. 4d, Supplementary Fig. 7a, b). The list of differentially expressed genes for all clusters is provided in Supplementary Data 3.

**Fig. 5:**
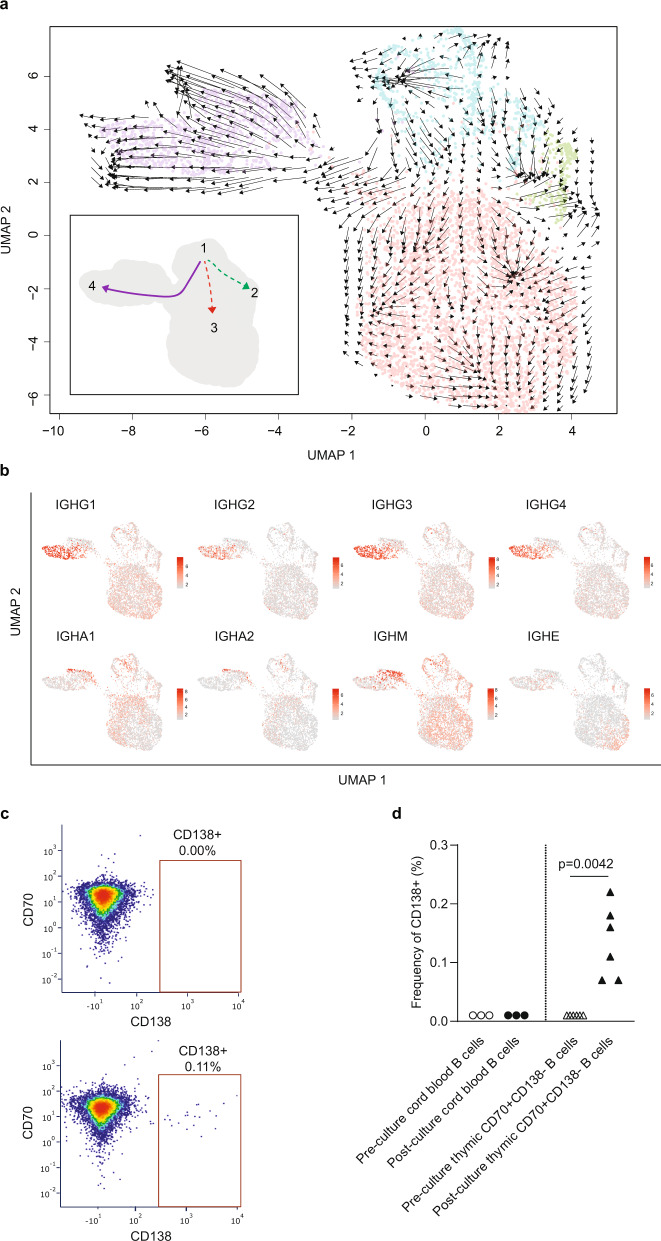
Intrathymic differentiation of thymic plasma cells in human neonates. **a** RNA velocity analysis of scRNA-seq data from thymic B cells and predicted cluster trajectory. **b** Expression of immunoglobulins in thymic individual B cells located mainly in Cluster 4 and colored by intensity from gray (no expression) to dark red (the highest expression). **c** Representative FACS plots of CD138^+^ plasma cells after in vitro culture of cord blood (top) and neonatal thymic (bottom) CD70^+^CD138^+^ B cells using conditions to promote differentiation into plasma cells. **d** Frequency of CD138^+^ plasma cells (%) pre and post-culture of CD70^+^CD138^−^ B cells isolated from cord blood (*n* = 3) or neonatal thymus specimens (*n* = 6). Two-sided *t*-test was performed.

RNA velocity describes the rate of gene expression change for individual genes at a given timepoint based on the ratio of its spliced and unspliced messenger RNA (mRNA)^32^. By applying RNA velocity, we predicted that the plasma cell cluster 4 originated from the highly proliferative cluster 1 (Fig. 5a). The relation between cluster 1 and clusters 2 and 3 was less evident (dotted lines in Fig. 5a). The trajectory analysis also revealed two different subgroups of plasma cells in destination cluster 4, expressing either all *IGH* subclass genes or *IGHA* and *IGHM* (Fig. 5a, b). This single-cell RNA velocity analysis provided supportive evidence that B cells differentiate into plasma cells, producing multiple classes and subclasses of immunoglobulins in the thymus of human neonates. These findings concur with the detection of IgM-, IgG-, IgA-, and IgE-ASC within neonatal CD21^−^CD35^−^ thymic B cells as reported in Fig. 1f.

To further confirm the ability of neonatal thymic B cells to differentiate into plasma cells we carried out in vitro differentiation assays. CD70^+^CD19^+^CD138^−^ B cells corresponding to cells in proliferating cluster 1 were purified by cell sorting from neonatal thymocytes or cord blood and cultured using previously reported conditions to induce differentiation of naïve B cells into plasma cells^33^. These conditions rely on a three-step process, the first of which is stimulation through the BCR. By omitting this first step, we assessed whether thymic B cells had previously been primed in vivo. As depicted in Fig. 5c, d, CD138^+^ plasma cells were successfully generated from neonatal thymic B cells, indicating that they had received prior stimulation in the thymus. In contrast, cord blood B cells were unable to differentiate into plasma cells under the same conditions.

### Thymic CD21^−^CD35^−^CD138^+^ show evidence of somatic hypermutation (SHM)

The generation of class-switched plasma cells is a central characteristic of B cell responses that typically occur in the germinal centers (GC) in a T-cell-dependent manner. Although, CSR can also take place outside of the GCs^34–36^, and without T cell help^37,38^ in what is known as extrafollicular response^39^. CSR is governed by activation-induced cytidine deaminase (AID) encoded by the *AICDA* gene expressed in CD21^−^CD35^−^ thymic B cells. AID is also responsible for the accumulation of SHM in differentiating cells as a means to diversify the antibody repertoire. We hypothesized that CSR of intrathymic B cells could also be accompanied by SHM and possible clonal selection. We conducted a comparative IGHV repertoire analysis of the two main thymic B cell subsets, CD21^+^CD35^+^ and CD21^−^CD35^−^ as well as differentiated plasma cells expressing CD138 within the CD21^−^CD35^−^ B cell subset (*n* = 5, Thymus 1, T1 to Thymus 5, T5; Supplementary Table 3).

**Fig. 6:**
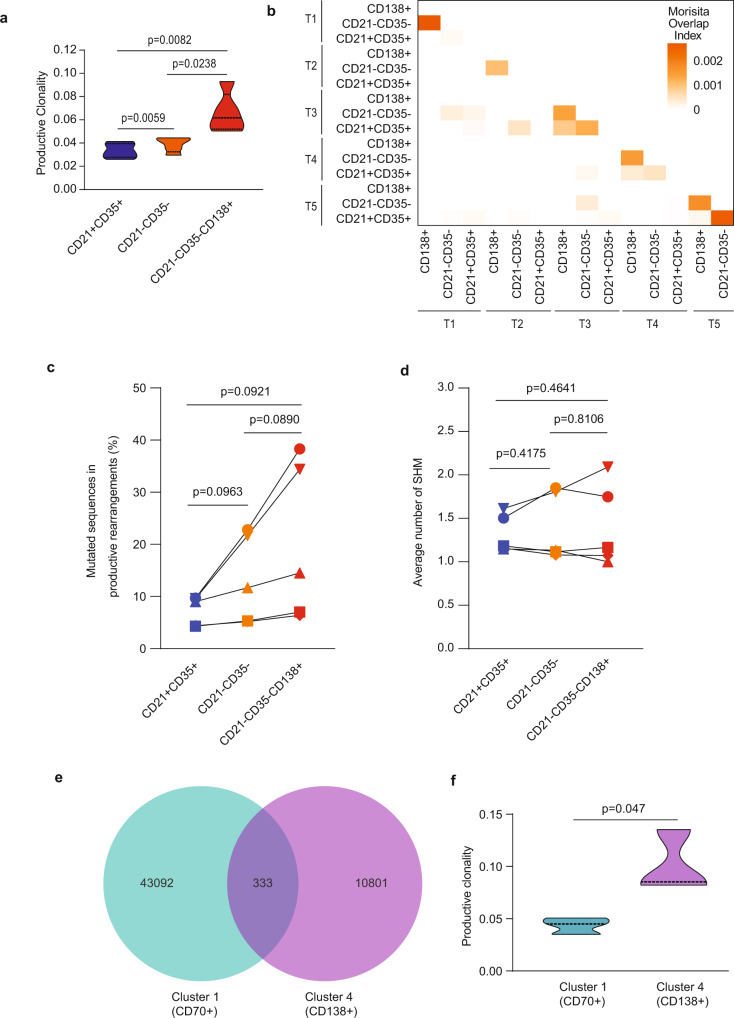
IGHV repertoire analysis of thymic B cells. **a** Productive clonality index in CD21^+^CD35^+^, CD21^−^CD35^−^, and CD21^−^CD35^−^CD138^+^ thymic B cell subsets (*n* = 5). **b** Morisita overlap index in CD21^+^CD35^+^, CD21^−^CD35^−^, and CD21^−^CD35^−^CD138^+^ thymic B cell subsets (*n* = 5) ordered by age from 1-day-old thymus (Thymus 1,T1) to 4-months-old thymus (Thymus 5, T5). **c** Percentage of mutated sequences within productive rearrangements in CD21^+^CD35^+^, CD21^−^CD35^−^, and CD21^−^CD35^−^CD138^+^ thymic B cell subsets (*n* = 5). **d** Average number of SHM in CD21^+^CD35^+^, CD21^−^CD35^−^, and CD21^−^CD35^−^CD138^+^ thymic B cell subsets (*n* = 5). **e** Venn diagram showing nucleotide IGHV sequences shared between Cluster 1 (CD19^+^CD70^+^CD138^−^) and Cluster 4 (CD19^+^CD138^+^) in the thymus of a representative donor (4 months old). **f** Productive clonality index of IGHV sequences of CD19^+^CD70^+^CD138^−^ (cluster 1) and CD19^+^CD138^+^ B cells (cluster 4) isolated from neonatal thymus specimens (age = 4 days, 1 months, and 4 months old, *n* = 3). Two-sided *t*-test was performed in all cases.

The IGHV repertoire diversity was evaluated using several clonality metrics. Higher clonality, Simpson clonality, and Simpson’s D indexes^40,41^ were found in CD21^−^CD35^−^ B cells compared to CD35^+^CD35^+^ B cells. Within CD21^−^CD35^−^ B cells, CD138^+^ cells had the highest clonality indexes in all cases (Fig. 6a, Supplementary Fig. 8a, b), suggesting clonal expansion within this cell population. In accordance with these findings, both the iChao1^42^ and Efron & Thisted^43^ estimators also indicated a lower diversity within CD138^+^ cells in 5 out the 5 thymus specimens (Supplementary Fig. 8c, d). A pairwise nucleotide sequence comparison using morisita index^44^ next revealed some limited level of sequence overlap between thymic CD21^−^CD35^−^ and CD138^+^ cells from the same thymuses but little if any overlap among these populations between the different thymuses (Fig. 6b). The frequency of IGHV rearrangements with somatic mutations appeared higher in thymic CD21^−^CD35^−^CD138^+^ plasma cells compared to total CD21^-^CD35^-^ or CD35^+^CD35^+^ B cells, although this difference was mostly observed for the 3- and 4-month-old thymus specimens, and did not reach statistical significance (Fig. 6c). The average number of SHM per mutated rearrangement was also very low (Fig. 6d). Lastly, the VH usage was similar across the thymuses and groups, with a predominance of rearrangements using IGHV3 and IGHV4 (Supplementary Fig. 8e, f).

We also used the IGHV repertoire analysis to assess the clonal correspondence between cells comprised in cluster 1 (CD19^+^CD70^+^CD138^−^) and cluster 4 (CD70^−^CD138^+^) as additional supportive evidence that plasma cells differentiate intrathymically. We hypothesized that cells in these two clusters share unique rearranged IGHV sequences, betraying their clonal relationship. As shown in Fig. 6e, Supplementary Fig. 9 and Supplementary Table 4, a significant fraction (~2.5%) of shared sequences was found between CD70^+^ and CD138^+^ subsets in the thymus of human neonates. A higher productive clonality was also found in CD138^+^ subset when compared with CD70^+^ subset (Fig. 6f). Together, these results suggested that a restricted contingent of B cells were selected to undergo differentiation into PC intrathymically.

### Neonatal thymic PC secrete immunoglobulins reactive to bacteria

**Fig. 7:**
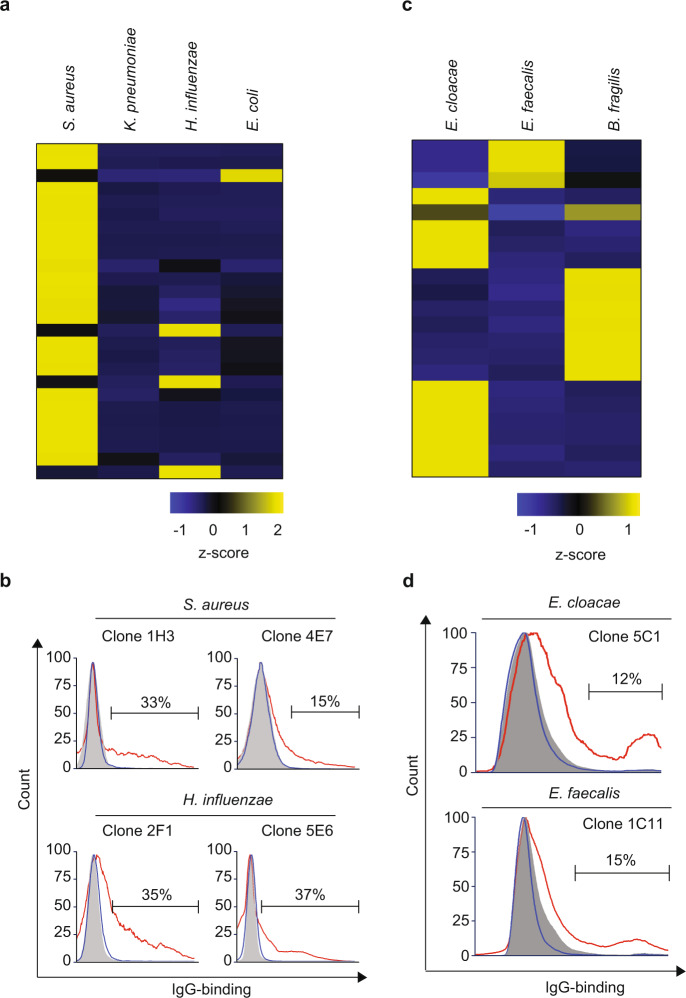
Reactivity of thymic plasma cells in human neonates to bacteria. **a** Heatmap representation of reactivity to *Staphylococcus aureus*, *Klebsiella pneumoniae*, *Haemophilus influenzae*, and *Escherichia coli*, of recombinant monoclonal antibodies generated from thymic plasma cells (*n* = 26). Results are expressed as normalized row z-score of percentage of antibody binding. **b** Histograms of representative monoclonal antibodies reactive to *S. aureus* (clones 1H3 and 4E7) and to *H. influenzae* (clones 2F1 and 5E6). The reactivity of the positive clones is shown with red lines. Nonreactive clones are shown with blue lines. Secondary antibody controls are shown with gray histograms. **c** Heatmap representation of reactivity to *Enterobacter cloacae*, *Enterococcus faecalis*, and *Bacteroides fragilis*, of recombinant monoclonal antibodies generated from thymic plasma cells (*n* = 20). Results are expressed as normalized row z-score of percentage of antibody binding. **d** Histograms of representative monoclonal antibodies reactive to *Enterobacter cloacae* (clone 5C1) and to *Enterococcus faecalis* (clone 1C11). The reactivity of the positive clones is shown with red lines. Nonreactive clones are shown with blue lines. Secondary antibody controls are shown with gray histograms.

Humans are born with a preset repertoire of protective natural antibodies (Nabs) assumed to develop without exposure to foreign antigen. A central characteristic of these antibodies is the ability to bind constitutive elements of the bacterial cell wall, explaining their antibacterial properties^45–47^. We hypothesized that thymic ASC differentiating perinatally could constitute a source of Nabs. To test this hypothesis, we generated recombinant monoclonal antibodies (rAbs) from 362 individual CD21^−^CD35^−^CD138^+^ isolated from five thymus specimens. All rAbs were then tested for their reactivity to pathogenic and commensal bacterial species, including *Staphylococcus aureus*, *Haemophilus influenzae*, *Klebsiella pneumoniae*, *Escherichia coli*, *Enterobacter cloacae*, *Enterococcus faecalis*, and *Bacteroides fragilis*. Between 2 and 15% of all PC clones reacted to at least one bacterial species (Fig. 7a–d, Supplementary Figs. 10, 11), with a predominance for reactivity to Gram-positive (*S. aureus and E. faecalis*) over Gram-negative bacteria. We did not detect any clones reactive to *K. pneumoniae* (Fig. 7a, Supplementary Fig. 10). Albeit limited, this screen underscores the frequency of plasma cells generated in the thymus of neonates and infants that secrete antibodies reactive to pathogenic and commensal bacteria.

**Fig. 8:**
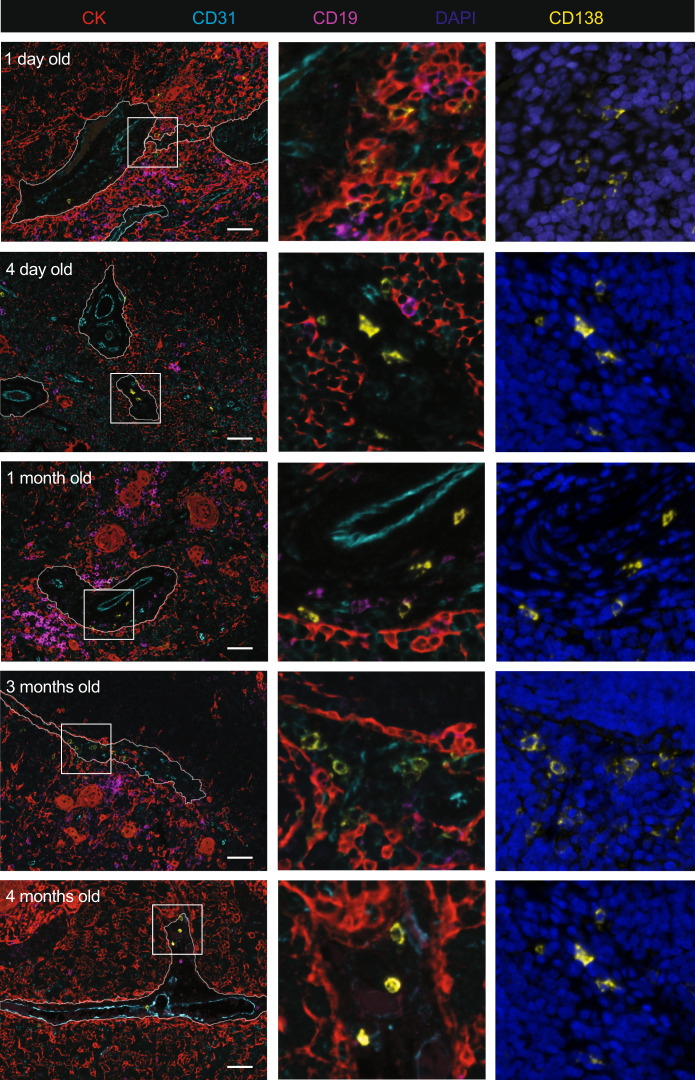
Location of thymic plasma cells in human neonates. Multiplex immunofluorescence of FFPE sections of five thymuses aged 1 day–4 months for thymic medullary marker pan-cytokeratin (red), endothelial marker CD31 (light blue), B cell marker CD19 (magenta), plasma cell marker CD138 (yellow) as well as nuclei staining with DAPI (dark blue). Framed sections correspond to ×20 magnification showing both all the cells and the location CD138^+^ plasma cells within the thymic perivascular space in human neonates. This experiment was repeated independently three times with similar results. Scale bar, 200 µm.

We have previously reported on the propensity of PC to accumulate in the thymic perivascular space (PVS) in infants and adults^6^. PC generated in neonates also appeared to locate around or in the PVS (Fig. 8). This specific location may reflect beneficial survival conditions in the PVS for ASC. Moreover, their proximity to vessels supports a contribution of these Nab-producing cells to peripheral immunity in neonates.

## Discussion

In this study we show that antibody-producing plasma cells are generated in the perinatal human thymus. This proposition is supported by the following lines of evidence: (1) The RNA velocity analysis linked proliferative B cells (cluster 1) to differentiated plasma cells (cluster 4), (2) a IGHV repertoire analysis revealed a shared clonal correspondence between cluster 1 and 4, (3) CD70^+^CD138^-^ thymic B cells in cluster 1 have the capacity to differentiate into CD138^+^ PC without the need for additional BCR stimulation, indicating that they have already received such stimulation in vivo, and (4) we could not detect any CD138^+^ cells in cord blood, arguing against the circulation of differentiated plasma cells from lymph nodes, spleen, or fetal liver to the thymus. Collectively, these data provide converging evidence to support the intrathymic differentiation of plasma cells in human neonates. Functional ASC were observed as early as one day after birth, indicating that the differentiation of these cells started during fetal life. A previous report using human fetal blood at various stages of gestation already provided evidence for CSR and SHM in utero in humans^48^. Our findings suggest that the thymus too  supports these fundamental aspects of B cell differentiation.

Our studies also revealed the important heterogeneity of thymic B cells in human neonates, attesting to B cell activity perinatally in this lymphoid organ. We first observed two main populations defined by the expression of the two main complement receptors CD21 and CD35. Based on their small size, structural features seen by TEM and lack of expression of activation markers, CD21^+^CD35^+^ B cells likely correspond to resting cells. In contrast, CD21^−^CD35^−^ B cells are larger, more diverse with respect to their molecular phenotype and express a host of activation and differentiation markers. Remarkably, activated CD21^low^ B cells had already been reported as a peripheral blood subset of activated B cells prone to further differentiation into plasmablasts and plasma cells following seasonal influenza vaccination^15^. In neonates, virtually all thymic CD21^−/low^ cells also lacked expression of CD35, the second complement receptor. Gene expression profiling indicated that the absence of CD21 and CD35 at the cell membrane was due to downregulation of the corresponding genes, rather than internalization of the receptors. Our studies examined whether human neonatal thymic B cells expressed genes included in the canonical B1 and B2 signature recently established in mice^18,19^. Strikingly, 6 out of 10 genes included in the mouse B1 transcriptional signature were upregulated in both thymic B cell subsets when compared to cord blood B cells. In contrast, none of the B2 genes were consistently upregulated in the same B cell subsets. Thymic B cells also displayed high expression of CD43, a putative marker of B1 cells^49,50^. It is important to note that some of the genes included in the B1 cell signature are not specific to B1 cells. While the similarities in gene expression profiles between the two cell types suggest that they share certain characteristics, these findings do not demonstrate that thymic B cells correspond to B1 B cells.

Within the heterogenous CD21^−^CD35^−^ thymic B cell population, our scRNAcell analysis revealed four distinct subset, the characteristics of which pointed to their possible functions. One subset corresponded to B cells undergoing active proliferation, most likely as the result of stimulation in situ. Based on RNA velocity cell trajectory analysis, this subset of dividing cells led to three destination subsets, suggesting that once stimulated, thymic B cells can differentiate into distinct functional phentotypes. The larger destination subset included cells expressing a number of co-stimulatory molecules previously ascribed to cell-to-cell interactions with T cells such as *CD80*, *CD86*, and MHC class II molecules. This subset likely includes B cells involved in T cell interaction^2–5,7^. B cells in the second destination subset shared the expression of *CD9* with IL-10-producing regulatory B cells^23,24^ as well as the expression of *EBI3*, a critical subunit of the inhibitory cytokine IL-35, with immunoregulatory plasma cells^25^. Moreover, cells within this subset also expressed the *CCL22* and *CCL17* genes encoding Th2 and Treg-actracting chemokines^21,22^. Specific expression of these two genes in IL-10-secretion Bregs was recently described in the context of antigen-induced arthritis in mice^51^. It is plausible that differentiated B cells within this subset also display suppressive capabilities and other functional characteristics of Bregs. The last destination cluster included ASCs characterized by a common expression signature of genes involved in differentiation into plasmablasts and plasma cells. Remarkably, the molecular profile of these cells together with the functional characterization of thymic ASC ex vivo, provided evidence of active CSR towards all classes and subclasses of immunoglobulins, including IgE, but excluding IgD. The physiological significance of such broad CSR processes is unclear.

B cells usually undergo CSR and differentiation into plasma cells upon antigen recognition and BCR stimulation. Several converging observations suggest that the differentiation of thymic B cells follow the same mechanism. First, GSEA indentified BCR signaling and B cell activation pathways as upregulated in thymic ASCs comparatively to other thymic B cells. Second, the higher clonality index observed in CD138^+^ cells compared to other thymic B cell subsets, supports the notion of clonal selection based on the specificity of the BCR. Taken together, these observations suggest that certain thymic B cell clones are selected to undergo differentiation into plasma cells on the basis of their BCR reactivity. As previously reported in mice, these may predominantly include autoreactive clones^2,5,9^.

Nabs present in the serum of nonimmunized animals and humans were recognized more than 100 years ago^52,53^. These antibodies are essential elements of natural immunity at birth, providing an innate line of defense against prevalent pathogens^45,54^. Children born with agammaglobulinemia or other forms of antibody deficiency invariably suffer from recurrent bacterial infections unless they receive immunoglobulin replacement therapy^55^. Our studies revealed that up to 15% of recombinant monoclonal antibodies generated from CD138^+^ thymic plasma cells also reacted to pathogenic Gram-negative and Gram-positive bacteria bacteria such as *H. influenzae* and *S. aureus*, respectively, as well as commensal bacteria, including *E. cloacae*. These cells may contribute to protective Nabs perinatally. Overall, our experiments describe the intrathymic differentiation of plasma cells secreting natural antibodies in human neonates. The identification of thymic PC clones reactive to pathogenic bacteria commonly infecting children born with primary antibody deficiencies could have important implications for the development of replacement therapies.

## Methods

### Sample collection

Thymuses were obtained from neonates and infants undergoing cardiac surgery at the NewYork- Presbyterian Hospital. Cord blood samples were obtained from Carolinas Cord Blood Bank (Duke University). Healthy buffy coats from adults were obtained from the New York Blood Center. All samples were discarded and deidentified according to the Protected Health Information (PHI) regulations, and considered non-human research samples. This study was approved by the Columbia University institutional review board.

### Sample processing

Thymic tissue was collected in cold phosphate-buffered saline (PBS) and washed extensively to remove the blood. A piece of tissue was kept on 10% paraformaldehyde solution for 24 h, then placed on 70% ethanol and finally paraffin embedded for histological analysis. The remaining tissue was homogenized using a gentleMACS tissue dissociator (Miltenyi Biotec) and filtered through a 40 µm cell strainer (BD Biosciences). Peripheral blood mononuclear cells (PBMCs) were isolated from cord blood and adult blood by Ficoll density gradient using Ficoll-PaquePLUS (GE HealthCare). Thymocyte and PBMC suspensions were frozen in heat inactivated fetal bovine serum (FBS) containing 10% dimethyl sulfoxide (DMSO, Fisher BioReagents) and kept in liquid nitrogen until use.

### Flow cytometry and cell sorting

Cryopreserved cells were thawed into warm RPMI media containing 10% FCS and washed in PBS. B cells were isolated by magnetic cell sorting with EasySep Human B Cell Enrichment Kit (Stem Cell Technologies) following the manufacturer’s instructions and stained in PBS with 2% FCS for 45 min with the following fluorochrome conjugated antibodies: anti-CD3 BV786 (clone SK7, BD Biosciences), anti-CD3 BV570 (clone UCHT1, Biolegend), anti-CD45 Qdot800 (clone HI30, Thermo Fisher Scientific), anti-CD19 PECy7 (clone HIB19, Tonbo Biosciences), anti-CD21 BV711 (clone B-ly4, BD Biosciences), anti-CD21 PECy5 (clone B-ly4, BD Biosciences), anti-CD21 V450 (clone B-ly4, BD Biosciences), anti-CD35 PE (clone E11, BD Biosciences), anti-CD35 FITC (clone E11, BD Biosciences), anti-CD38 BV650 (clone HIT2, Biolegend), anti-CD138 PE (clone 44F9, Miltenyi Biotec), anti-CD138 VB515 (clone 44F9, Miltenyi Biotec), anti-CD70 APC (clone 113-16, Biolegend), anti-CD27 APC Cy7 (clone O323, Tonbo Biosciences), anti-IgG Alexa Fluor 700 (clone G8-145, BD Biosciences), anti-IgM BV421 (clone MHM-88, Biolegend), anti-IgA APC (clone IS11-8E11, Miltenyi Biotec), anti-IgE BV480 (clone G7-26, BD Biosciences), anti-IgD BV510 (clone IA6-2, BD Biosciences), anti-CD69 PECy5 (clone FN50, Biolegend), anti-CD80 BV711 (clone 2D10, Biolegend), anti-CD86 Alexa Fluor 647 (clone IT2.2, Biolegend), anti-PD1 PE-Dazzle594 (clone EH12.2H7, Biolegend), anti-CD39 BV650 (clone TU66, BD Biosciences), anti-CD59 PE (clone H19, Biolegend), anti-CD269 PerCPCy5.5 (clone 19F2, Biolegend), anti-XBP1S PE (clone Q3-695, BD Biosciences), anti-IRF4 PerCPCy5 (clone IRF4.3E4, Biolegend), anti-BLIMP1 Alexa Fluor 647 (clone 6D3, BD Biosciences), anti-Ki67 FITC (clone SolA15, Thermo Fisher Scientific). Antibody dilutions are listed in Supplementary Table 5.

For intracellular staining, cells were fixed and permeabilized using Transcription Factor Staining Buffer Set (eBioscience) following the manufacturer’s instructions prior to staining. Cells were washed in cold PBS with 2% FCS, filtered through a 70 µm cell strainer and acquired using BD LSRFortessa or Cytek Aurora flow cytometer. Data were analyzed using FCS Express 6 Research Edition (DeNovo Softaware).

### ELISpot assay

To assess the frequency of spontaneous antibody-secreting cells in the thymus of newborns or in cord blood, ELISpot was carried out following the manufacturer’s instructions with some modifications. Briefly, ELISpot plates (MSIPS4510, Millipore-Sigma) were coated with anti-human IgG (7.5 µg ml^−1^, Mabtech), anti-human IgM (5 µg ml^−1^, Mabtech), anti-human IgA (5 µg ml^−1^, Mabtech), and anti-human IgE (5 µg ml^−1^, Mabtech). After overnight coating and blocking with 1% FCS-PBS for 30 min, total thymocytes, PBMCs from cord blood or sorted thymic B cells (CD19^+^CD21^+^CD35^+^ and CD19^+^CD21^−^CD35^−^) were plated for detection of total ASCs. After overnight incubation at 37 °C, bound antibodies were detected using biotinylated anti-human IgG (1 µg ml^−1^, Mabtech), anti-human IgM (1 µg ml^−1^, Mabtech), anti-human IgA (1 µg ml^−1^, Mabtech), and anti-human IgE (1 µg ml^−1^, Mabtech). Spots were developed with ELISPOT Blue Color Module (R&D System) using streptavidin-conjugated alkaline phosphatase and 5-bromo-4-chloro-3-indolyl phosphate (BCIP)/nitro blue tetrazolium (NBT) as substrates. Spots were quantified using EazyReader software version 18.9 in the ELISpot Bioreader 5000 (BioSys).

### Transmission electron microscopy

CD19^+^CD21^+^CD35^+^ and CD19^+^CD21^−^CD35^−^ cells were sorted on a BD FACS Aria cell sorter and placed in 2% paraformaldehyde/2.5% glutaraldehyde in 0.1 M sodium cacodylate fixative buffer, post-fixed with 1% osmium tetroxide followed by 2% uranyl acetate, dehydrated through a graded series of ethanol and embedded in LX112 resin (LADD Research Industries, Burlington, VT). Ultrathin sections were cut using a Leica Ultracut UC7 ultramicrotome (Leica Microsystems), stained with uranyl acetate followed by lead citrate. Grids were examined on a JEOL 1400EX transmission electron microscope at 120 kV. Images were acquired using Gatan Microscopy Suite Software version 2 (Gatan). Images were analyzed using ImageJ software version 1.52a (NIH, USA, https://imagej.nih.gov/ij/).

### RNA sequencing

Thymic B cells (CD19^+^CD21^+^CD35^+^ and CD19^+^CD21^−^CD35^−^), cord blood and adult blood CD19^+^B cells were sorted as mentioned above and place in lysis buffer (Qiagen). Total RNA extraction was performed RNAeasy Micro Kit (Qiagen) according to the manufacturer’s instructions. RNA QC was done using TapeStation Analysis Software version A.02.02 (Agilent Technologies). Library preparation was performed using the NEBNext Ultra RNA Library Preparation kit with PolyA selection workflow. Libraries were sequenced on a Illumina HiSeq 4000 using a 2 × 150 bp Paired End lengths. Raw sequence data generated was converted into fastq files and demultiplexed using Illumina’s bcl2fastq version 2.17. Raw reads QC was performed using FASTQC. Reads were aligned using STAR aligner v2.5.2b to map the reads to the GRCh38 reference human genome. RNA-sequencing data analysis was performed using R Studio version 3.6.0^56^ with *sva* package version 3.40.0 for batch correction using a linear model^57^ and DESEeq2 package version 1.30.0 for differential expression analysis^58^. Plots were generated using *ggplot2* package version 3.3.3^59^. Geneset enrichment pathways analysis was performed using GSEA software version 4.0.1^60,61^.

### Proteomics

Sorted CD19^+^CD21^+^CD35^+^ and CD19^+^CD21^−^CD35^−^ cells were analyzed using in-StageTip (iST) method according to the manufacturer’s instructions^62^. Cells were lysed in lysis buffer (8 M Urea, 10 mM TCEP, 40 mM CAA, 100 mM Tris pH 8.5), and digested with LysC/trypsin overnight at 37 °C within a iST reactor. Next, peptides were eluted into vials and dried using SpeedVac. Dried peptides were dissolved in acetonitrile/formic acid buffer before being submitted to MS/MS on the Orbitrap Fusion Tribrid mass spectrometer. The MS files were identified and analyzed using MaxQuant software package version 2.0.1.0 and the Perseus software platform version 1.6.15.0 was used for statistical analysis. Results are expressed as label-free quantification (LFQ) intensity. Statistical differences were assigned when *p* < 0.05.

### Single-cell RNA sequencing

Single-cell RNA sequencing of sorted CD21^−^CD35^−^CD19^+^ thymic B cells was conducted using Chromium Single Cell 3′ Reagent Kits v2 (10X Genomics) according to the manufacturer’s instructions. The libraries were quantified using KAPA hgDNA Quantification and QC Kit (Kapa Biosystems) and sequenced via NovaSeq 6000 (Illumina). Following the sequencing, the raw data from each sample were demultiplexed, aligned to the GRCh38-1.2.0. human reference genome, and UMI counts were quantified using the 10X Genomics Cell Ranger pipeline (v2.1.1, 10X Genomics). Data analysis was then continued with the filtered barcode matrix files using the Seurat package version 3.0^63^ in R version 3.6.0^56^. Donor samples were sequenced in two batches [batch#1: donor 72 4 month old (4mo) and donor 127 (4 day); batch#2: donor 131 (4 day), donor 73 (4 day), donor 29 (4mo), and donor 135 (4mo)]. For the initial QC step, we filtered out dead and doublet cells that express high percentage of mitochondrial and cellular genes respectively. For donors 72 and 127 we removed any cell that expressed >4% mitochondrial transcripts and cells that expressed <200 or >4000 genes, for donors 131 and 73 we removed any cell that expressed >15% mitochondrial transcripts and cells that expressed <200 or >5000 genes, for donor 29 and 135 we removed any cell that expressed >15% mitochondrial transcripts and cells that expressed <200 or >6000 genes. We then used SCTransform function of Seurat package to pre-process (regressing out percent.mt and nCount_RNA), normalize and scale gene expression for each of the sample individually and identify the most variable genes for each individual sample^64^. We then ran PCA for each sample separately and used the top 30 principal components (PCs) to run UMAP and cluster the cells of each individual sample into subpopulations using Seurat’s implementation of a shared nearest neighbor modularity optimization-based clustering algorithm (Louvain’s original algorithm). We removed any non-B cell clusters identified by this approach and then renormalized and scaled the data using the SCTransform function. Following QC, we got 2745 cells from donor 72, 2563 cells from donor 127, 4534 cells from donor 131, 4492 cells from donor 73, 4212 cells from donor 29, and 5511 cells from donor 135.

The top 3000 highly variable genes from each sample were used to generate the anchor genes list used to integrate the six samples as described in the Seurat package ScTransform data integration workflow (https://bit.ly/32GMckR). We ran PCA on the integrated dataset of 24,057 cells and used the top 30 significant PCs for running UMAP and clustering analysis. The cells were clustered into subpopulations using Seurat’s implementation of a shared nearest neighbor modularity optimization-based clustering algorithm (Louvain’s original algorithm). We compared various cluster resolutions (0.0–1.0) and used cluster mapper to identify optimal cluster resolution that yields biologically relevant cell clusters. Cell clusters were visualized using UMAP^65^. For differential gene expression, we used model-based analysis of single-cell Transcriptomics (MAST) test using MAST package version 1.18.0^66^ (log fc ≥ 0.25) and only selected the genes with adjusted *p*-value based on Bonferroni correction <0.05 were used for further GSEA analysis (as described above). We merged clusters that had fewer that 10 differentially expressed genes to finally yield four distinct cell clusters with distinct biological functions. RNA velocity analysis was performed using Velocyto pipeline^32^ and integrated into the Seurat analysis.

### In vitro differentiation of thymic B cells into plasma cells

Thymic B cells from cluster 1 were differentiated into plasma cells in vitro using a modified version of the three-steps culture system^33^. In our in vitro model, the first step that typically consists of B cell activation was omitted. Sorted CD19^+^CD70^+^CD138^−^ thymic and cord blood B cells were directly culture in RPMI supplemented with 10% FCS, 2-Mercaptoethanol (55 µM, Life Technologies), IL-2 (50 ng ml^−1^, Preprotech), IL-6 (50 ng ml^−1^, Preprotech), IL-10 (50 ng ml^−1^, Preprotech), and IL-15 (25 ng ml^−1^, Preprotech). After 3 days, the supplemented RPMI media was replace with another cocktail containing IL-6 (50 ng ml^−1^, Preprotech), IL-15 (25 ng ml^−1^, Preprotech), and IFNα−2b (10^5^ units ml^−1^, PBL Assay Science). After another 3 days, the presence of differentiated CD138^+^ plasma cells was evaluated by flow cytometry as described above.

### BCR sequencing

CD19^+^CD21^+^CD35^+^, CD19^+^CD21^−^CD35^−^, CD19^+^CD21^−^CD35^−^CD138^+^, CD19^+^CD70^+^, and CD19^+^CD138^+^ cell populations were sorted as described above. DNA extraction was performed using DNeasy Blood and Tissue Kit (Qiagen) following the manufacturer’s instructions and sample was eluted in TE buffer. IGHV sequencing was performed by Adaptive Biotechnologies using the ImmunoSEQ survey level assay. The assay uses 86 primers for the IGHV gene segment, 15 primers for the IGHD gene segment, and 7 primers for the IGHJ gene segment^67^. This generated a fragment capable of identifying the entire spectrum of unique VDJ combinations including functional genes, pseudogenes, and open reading frames. Next, amplicons were sequenced using the Illumina HiSeq platform. The resulting 130 bp sequences permitted inference of the corresponding germline sequences^67^, and are denoted IGH–VDJ transcripts. A suite of custom algorithms has been developed by Adaptive Biotechnologies to verify, collapse, align and catalog the CDR3 sequences. To assess and remove PCR bias from the multiplex PCR assay, a synthetic immune system with all possible V–J combinations was precisely quantitated as described elsewhere^68^. The data were subsequently analyzed and visualized using the ImmunoSEQ analyzer provide by Adaptive Biotechnologies. A series of metrics and indexes were applied to the IGHV repertoire results to evaluate and compare the diversity of goups of sequences (rearrangements) between the different subsets of thymic B cells^40–43^. Clonality values near 0 indicate the highest diversity, whereas values close to 1 represent samples near monoclonality. Clonal overlap was calculated using only productive rearrangements. Nucleotide sequences with 100% identical CDR3 were attributed to the same clones. Results were plotted as a Venn Diagram using the ImmunoSEQ Analyzer version 3.0.

### Recombinant antibody cloning and expression

CD19^+^CD21^+^CD35^+^, CD19^+^CD21^−^CD35^−^, and CD19^+^CD21^−^CD35^−^CD138^+^ thymic cells were sorted as previously mentioned into 384-well plates filled with hypotonic lysis buffer^69,70^ containing 10 nM TRIS and 0.75 units ml^−1^ of RNASin plus (Qiagen) at 4 °C and stored at −80 °C. Next, cDNA was generated using the High-capacity cDNA generation kit (Applied Biosystems) following the manufacturer’s instructions. Multiplexed PCR was performed using primers specific for *IGHV*, *IGKV*, *IGHC*, *IGKC*, and *IGLC* gene segments. The list of primers is in Supplementary Table 6. Resulting amplicons were used as templates for semi-nested PCR to isolate the heavy and light chain genes and to incorporate modifications to allow ligation independent cloning into expression vectors^71^. Resulting amplicons were inserted into mammalian expression plasmids containing the *IGG1 IGKC*, or *IGLC* gene sequence. Recombinant antibodies were then generated by co-transfection of plasmids encoding Ig heavy and light chain pairs into 293FS cells (Invitrogen) using standard polyethylenimine (PEI) transfection methods^72^. For each clone, 0.5 µg of each heavy chain and light chain plasmid were combined with 3 µg of PEI (1 mg ml^−1^ in H_2_O pH 7.0) in 293FS media (Invitrogen). Each sample was incubated for 10 min at room temperature and then added into the wells containing 10^6^ cell ml^−1^ and incubated for 5 days shaking at 37 °C in the incubator.

### Quantitation of Ig clone supernatants

The concentration of IgG in the supernatant of 293FS cells transfected with plasmids encoding Ig heavy and light chain pairs was assessed by ELISA using the Human IgG ELISA Quantitation kit (E80-104, Bethyl Laboratories) following the manufacturer’s instructions. Optical density was read at 450 nm using BioTek Synergy H1 plate reader. Concentration was calculated based on the standard curve for human reference serum and expressed in ng per ml.

### Bacterial culture

*Staphylococcus aureus* GP22, *Escherichia coli* (ATCC 25922), *Klebsiella pneumoniae* KP35^73^, were picked from plated colonies and grown in an overnight culture of 2 mL TSB at 37 °C. *Haemophilus influenzae* (ATCC 19418) was grown in a lawn on chocolate plates overnight at 37 °C. *Enterobacter cloacae* KP1117^74^ was picked from plated colonies and grown in an overnight culture of 2 mL tryptic soy broth (TSB) at 37 °C. *Enterococcus faecalis* NR 7112^75^ was grown in a lawn on chocolate plates overnight at 37 °C. *Bacteroides fragilis* (ATCC 25285) was prepared in a vinyl anaerobic chamber (Coy Labs #032714). *B. fragilis* was inoculated from a monoclonal stock into 5 mL of Gifu-Anaerobic-Broth-GAM (HiMedia) and grown overnight. This isolate was then subcultured in Gifu-Anaerobic-Broth-GAM and grown until the OD reached ~0.35. Bacterial cultures were subcultured or resuspended in TSB until the OD was ~0.35 and 10 mL of these suspensions were centrifuged at 4000 × *g* at 4 °C. Pellets were resuspended in TSB at 10^9^ CFU ml^−1^.

### Bacterial reactivity of IgG clone supernatants

IgG clone supernatants were assessed for reactivity to seven relevant bacterial species (see above). Briefly, each IgG clone supernatant was incubated with each bacterial culture for 30 min at 37 °C in 96-well cell culture plates (Corning Incorporated). After washing, bacteria were incubated with FITC-conjugated anti-human IgG (dilution 1:200, A24477, Fisher Thermo Scientific) for 30 min at RT. After washing, bacteria were fixed in 10% formalin and acquired on a BD LSRFortessa flow cytometer with high-throughput sampling capabilities. Flow cytometry file data were analyzed as described elsewhere in this paper.

### Immunofluorescence of paraffin-embedded thymus sections

Tissue sections were deparaffinized in xylene for 10 min, washed with 100% ethanol followed by 95, 80, 70, and 50% ethanol, and then rinsed in distilled water. Samples were processed for antigen retrieval, blocking and staining following the Opal Multiplex IHC protocol (PerkinElmer) as described elsewhere^76^. Anti-CD19 (clone BT51E, NCL-L-CD19-163, Leica Biosystems), anti-CD31 (clone C31.3 + JC/70 A, ab199012, Abcam), anti-cytokeratin (clone PCK-26, ab6401, Abcam), and anti-CD138 (clone MI15, PA0088, Leica Biosystems) were used as primary antibodies. Opal 7-Color IHC Kit (PerkinElmer) was used as conjugated antibodies as described by the manufacturer’s instructions. Antibody dilutions are listed in Supplementary Table 6. Finally, after DAPI staining, slides were mounted with VECTASHIELD antifade mounting media (Vector Laboratories). Images were taken using Vectra 3.0 Automated Quantitative Pathology Imaging System (PerkinElmer) and InForm cell analysis software version 2.4.6 (PerkinElmer). Images were evaluated and validated by an experienced pathologist.

### Statistical analysis

The results are presented as mean ± SD and/or normalized z-score unless otherwise specified in the figure legends. Population size is described in the figure legend. All the statistical analyses were performed using GraphPad Prism software version 7.0. Differences were considered statistically significant when *p* < 0.05 using paired/unpair *t*-test. For RNA-seq, differences were considered statistically significant when Benjamini–Hochberg adjusted *p*-value > 0.05 and absolute log_2_ fold change > 1. Results of statistical tests are listed in Supplementary Data 1, 2, and 3.

### Reporting summary

Further information on research design is available in the Nature Research Reporting Summary linked to this article.

## Supplementary information

Supplementary Information

Description of Additional Supplementary Files

Supplementary Data 1

Supplementary Data 2

Supplementary Data 3

Reporting Summary

## Data Availability

The processed data and transcriptome datasets for both RNA-Seq and Single-Cell RNA-Seq generated during this study are available at the NCBI Gene expression Omnibus with the accession number: GSE152453 and GSE153117, respectively. The database for the GRCh38 [https://useast.ensembl.org/Homo_sapiens/Info/Annotation] (GCA_000001405.28) human reference genome is available online. DNA sequencing data generated for BCR repertoire analysis are available at the NCBI Sequence Read Archive with the accession number PRJNA761408. Suppementary Data files are provided with the online version of this paper. Source data are provided with this paper.

## Source data

Source Data

## Code availability

Codes for both bulk RNA sequencing and single-cell RNA-sequencing analyses are available on GitHub (https://github.com/Dr-Cordero/natcomms-2021).
