# Supplementary material for: Intrathymic differentiation of natural antibody-producing plasma cells in human neonates
Source: Nat Commun. 2021 Oct 1;12:5761. doi: 10.1038/s41467-021-26069-2 (PMC8486820; doi:10.1038/s41467-021-26069-2)
Supplement: Supplementary file 1 — Supplementary Information [file 41467_2021_26069_MOESM1_ESM.pdf]

Intrathymic differentiation of natural antibody-producing plasma cells in human neonates

Hector Cordero[1], Rodney G. King[2], Pranay Dogra[1, 3], Chloe Dufeu[1], Sarah B. See[1], Alexander M. Chong[4], Anne-Catrin Uhlemann[4], Siu-Hong Ho[1], David M. Kalfa[5], Emile A. Bacha[5], John F. Kearney[2], Emmanuel Zorn[1*]

[1] Columbia Center for Translational Immunology, Columbia University Medical Center, New York 10032 NY, United States.

[2] Department of Microbiology, University of Alabama at Birmingham, 35294 AL, United States.

[3] Department of Microbiology and Immunology, Columbia University Medical Center, New York 10032 NY, United States.

[4] Division of Infectious Diseases in the College of Physicians and Surgeons, Columbia University Medical Center, New York 10032 NY, United States.

[5] Division of Cardiac, Thoracic and Vascular Surgery, Columbia University Medical Center, New York 10032 NY, United States.

Supplementary Information

Supplementary Figures 1-11

Supplementary Tables 1-6

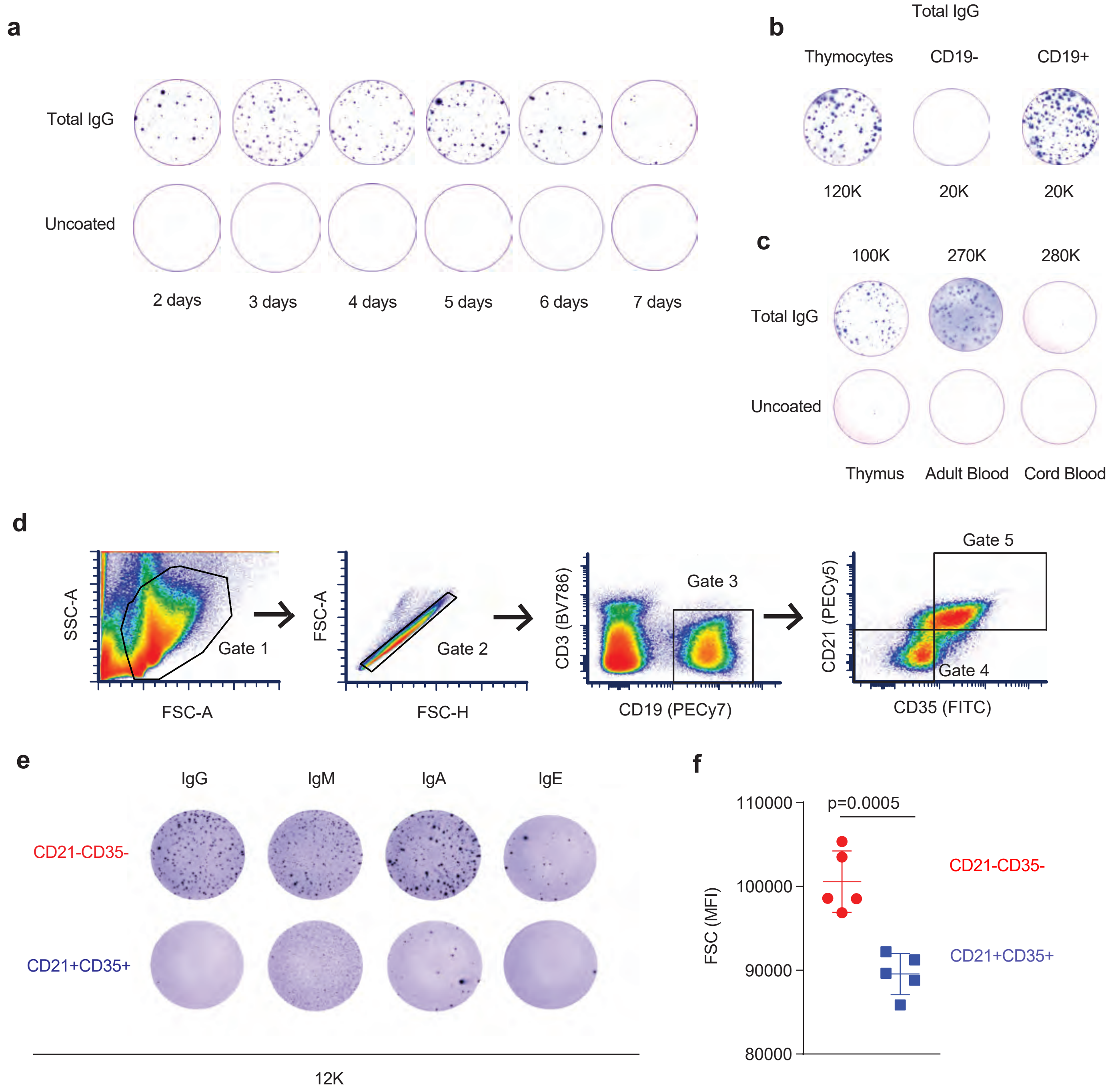

Supplementary Fig. 1. The human neonate thymus contains antibody-secreting cells comprised within the CD19+CD21-CD35- B cell subset. a Representative ELISPOT result showing IgG-ASCs within thymocytes during the first week of life. Between 100K-400K thymocytes were coated in each well. **b** Representative ELISPOT results showing IgG-ASC in total, CD19- and CD19+ thymocytes. **c** Representative ELISPOT results showing IgG-ASC within thymus, adult and cord blood CD19+ B cells. **d** FACS gating strategy used in Figure 1-6 to obtain human thymic B cell subsets, CD21-CD35- and CD21+CD35+. **e** Representative ELISPOT results showing IgG, IgM, IgA and IgE-ASCs in CD21-CD35- and CD21+CD35+ thymic B cells. **f** Forward scatter (size) of sorted CD19+CD21-CD35- andCD19+CD21+CD35+ thymic B cells measured by flow cytometry (n=5). Bars are defined as mean +/- standard deviation (SD). Two-sided t-test was performed.

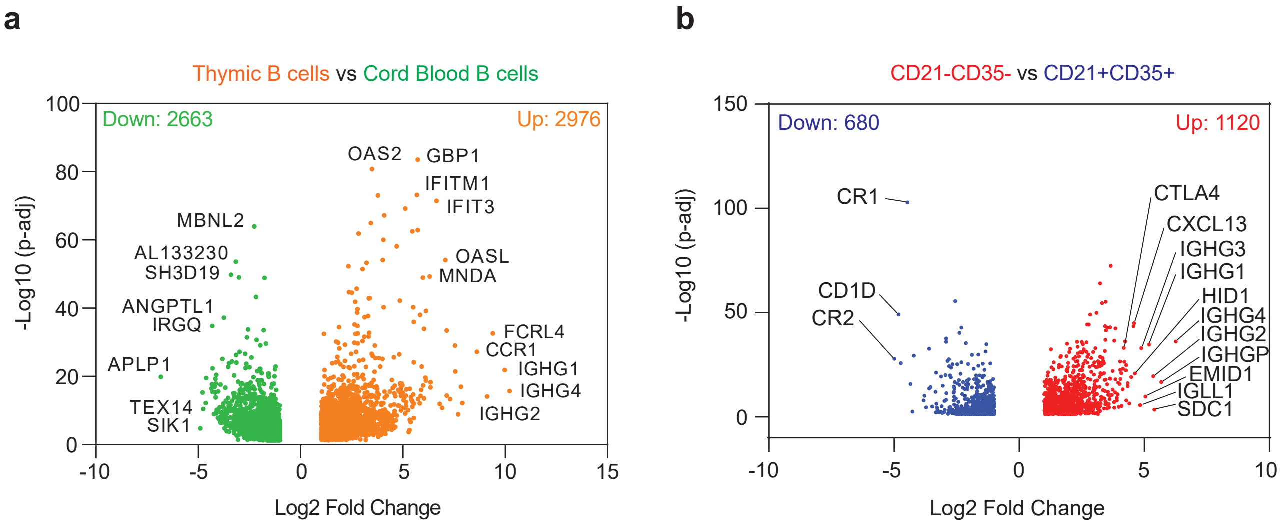

Supplementary Fig. 2. Transcriptome profiling of cord blood B cells and thymic CD21-CD35- and CD21+CD35+ B cell subsets in human neonates. **a** Volcano plot of DE genes between thymic B cells and cord blood B cells (DESeq2, two-sided Wald test with Benjamini-Hochberg false discovery ration (FDR) adjustment, p-adj < 0.05 and log2 fold change < 1). **b** Volcano plot of DE genes between both thymic CD21-CD35- and CD21+CD35+ B cell subsets (DESeq2, two-sided Wald test with Benjamini-Hochberg false discovery ration (FDR) adjustment, p-adj < 0.05 and log2 fold change < 1).

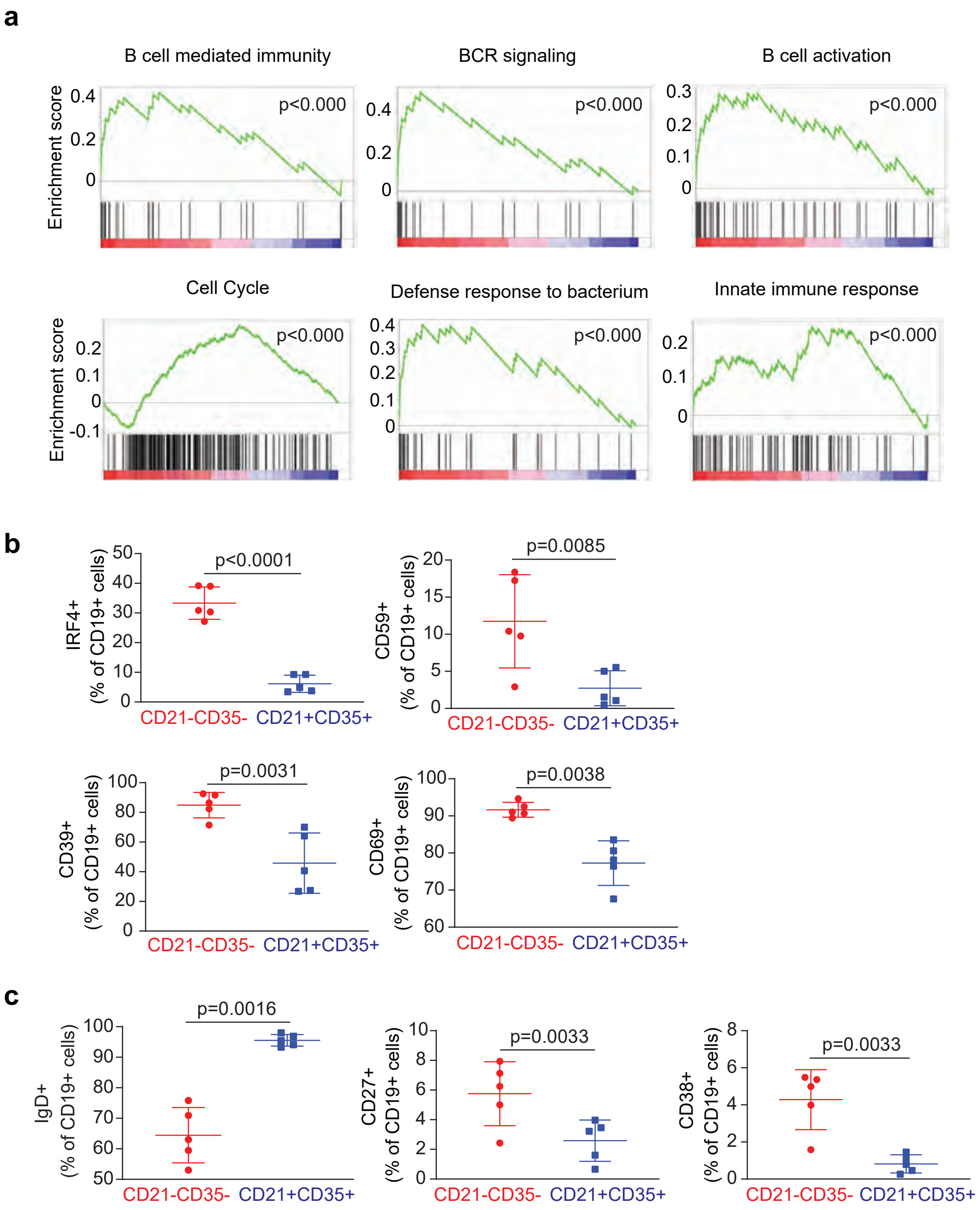

Supplementary Fig. 3. GSEA and surface markers analysis of CD21-CD35- and CD21+CD35+ B cell subsets in the thymus of neonates. a Selected gene set enrichment analysis (GSEA) plots in CD21-CD35- and CD21+CD35+ B cell subsets in the thymus of neonates using GSEA software version 4.0.1 from Broad Institute (https://bit.ly/2PXIzzY). Two-sided Kolmogorov–Smirnov test. **b** Frequency of IRF4+, CD59+, CD39+ and CD69+ subsets within CD19+CD21-CD35- and CD19+CD21+CD35+ in the thymus of neonates and infants aged 1 day to 4 months, measured by flow cytometry (n=5). Bars are defined as mean +/- standard deviation (SD). Two-sided t-test was performed. **c** Frequency of IgD+, CD27+ and CD38+ subsets within CD19+CD21-CD35- and CD19+CD21+CD35+ in the thymus of neonates and infants aged 1 day to 4 months, measured by flow cytometry (n=5). Bars are defined as mean +/- standard deviation (SD). Two-sided t-test was performed.

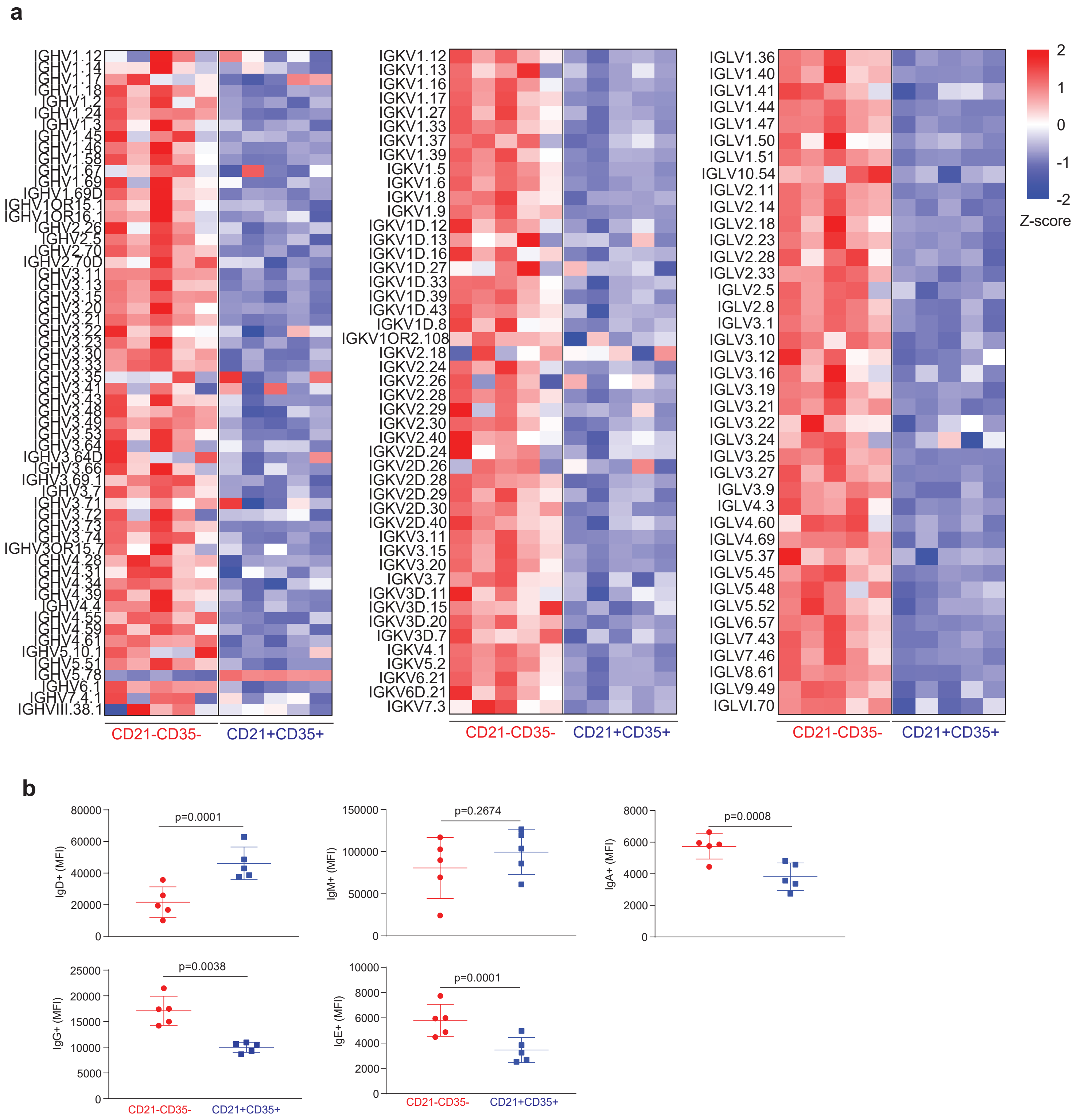

Supplementary Fig. 4. Immunoglobulins and variable region genes transcripts in CD21-CD35-and CD21 +CD35+ thymic B cell subsets of human neonates. a Heat map representation of transcripts of variable region genes coding for heavy, kappa and light immunoglobulin chains in CD19+CD21-CD35-and CD19+CD21+CD35+ thymic B cells. Data are expressed as normalized row Z-score of log values (n=5). Wald-test with Benjamini-Hochberg false discovery ration (FDR) adjustment. **b** Membrane expression of IgD+, IgM+, IgA+, IgG+ and IgE+ in CD21-CD35- and CD21+CD35+ thymic B cell subsets measured by flow cytometry (n=5). Bars are defined as mean +/- standard deviation (SD). Two-sided t-test was performed.

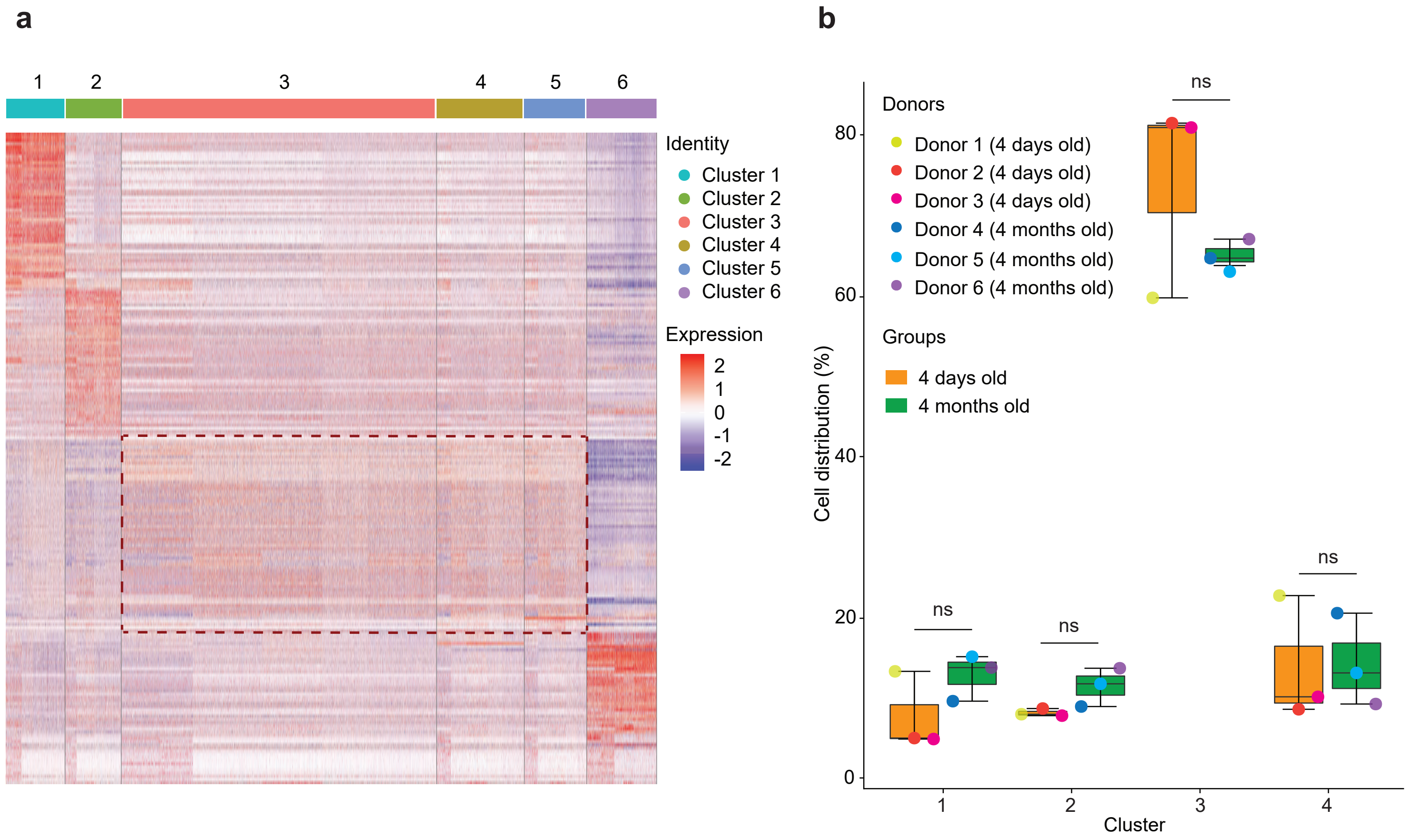

Supplementary Fig. 5. Gene expression and cell distribution by age. a Heat map representation of top 30 genes expressed in 6 clusters of thymic CD19+CD21-CD35- B cells based on single cell RNA sequencing analysis of thymic B cells. The dashed box highlights the similar expression of clusters 3, 4 and 5. Results are expressed as normalized row z-score of log values. Two-sided MAST (Model-based Analysis of Single-cell Transcriptomics) test with Bonferroni correction was performed. **b** Cell distribution plots per B cell cluster (%) in six different thymuses across two different groups of age, 4 days old (orange, n=3 biologically independent samples) and 4 months old (green, n=3 biologically independent samples) in the scRNA-seq analysis. Centre is median, bounds are 25th and 75th percentiles, the edges of the whiskers are the minimum and maximum possible values.

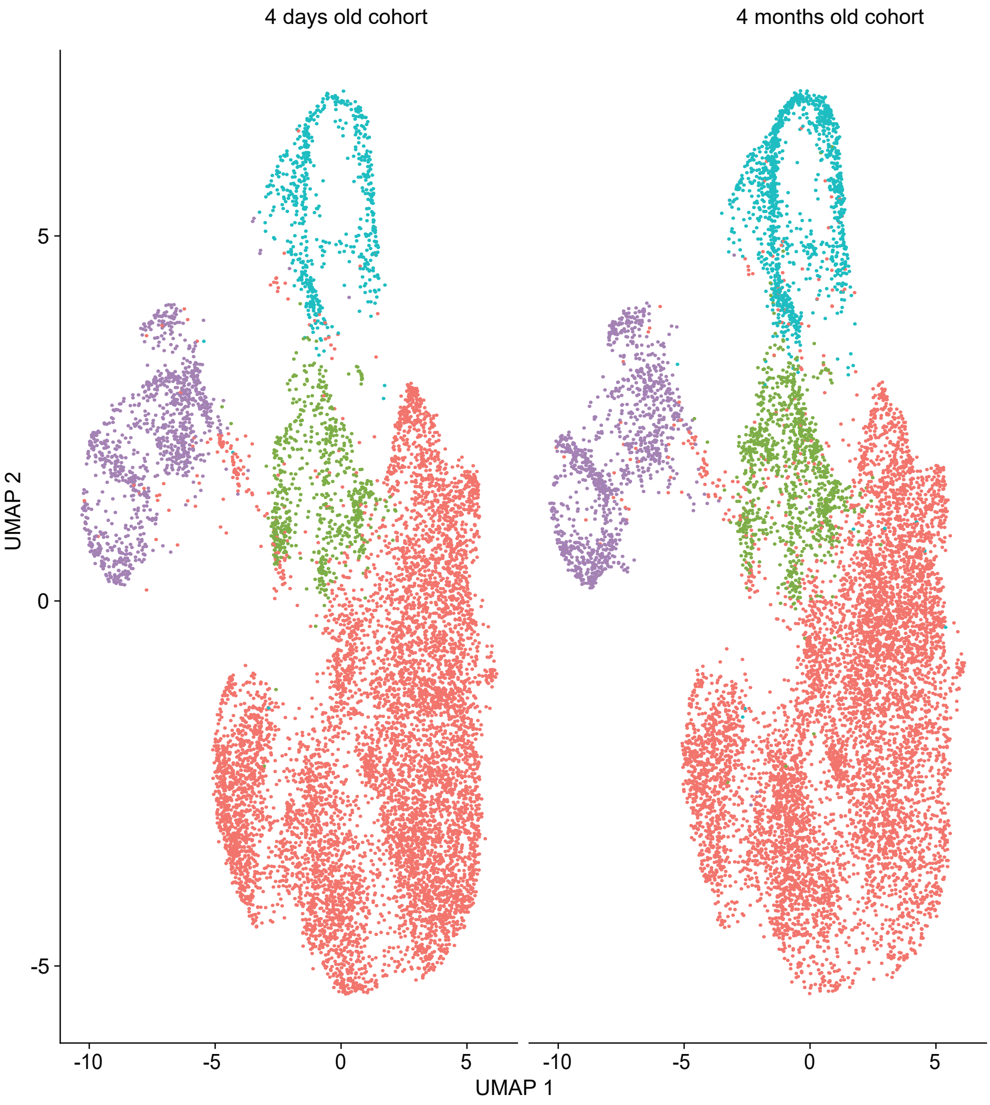

Supplementary Fig. 6. UMAP comparison by age. UMAP embeddings of integrated scRNA-seq data from thymic CD19+CD21-CD35- B cells showing the cell distribution in 4-day-old (n=3) and 4-month-old thymus specimens (n=3).

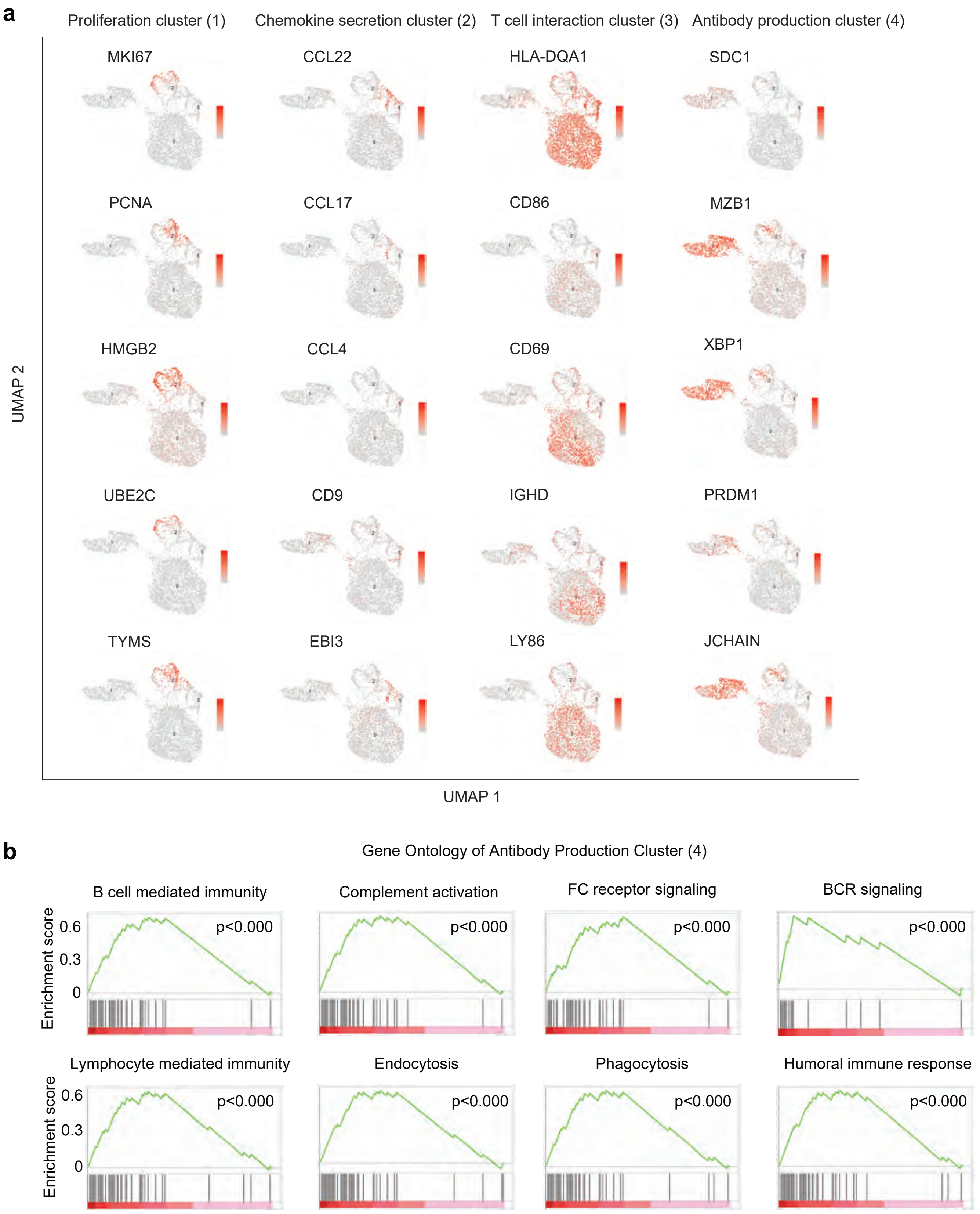

Supplementary Fig. 7. Single cell sequencing of thymic B cells in human neonates. a Expression of key markers defining each cluster of thymic B cells and colored by intensity from gray (no expression) to dark red (highest expression). Two-sided MAST (Model-based Analysis of Single-cell Transcriptomics) test with Bonferroni correction across gene dataset. **b** Selected gene set enrichment analysis (GSEA) plots in Antibody Production Cluster using GSEA software version 4.0.1 from Broad Institute (https://bit.ly/2PXIzzY). Two-sided Kolmogorov–Smirnov test.

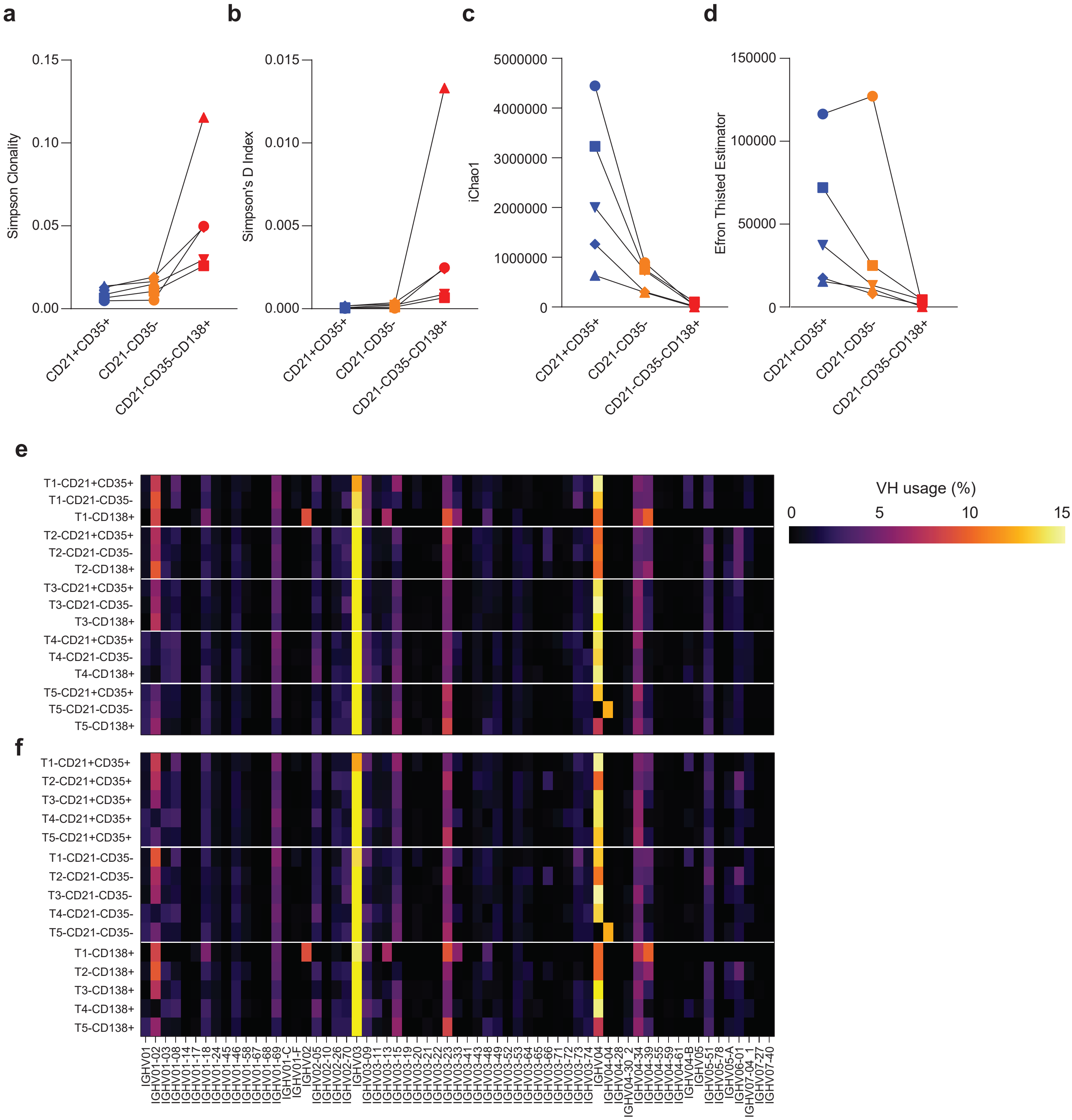

Supplementary Fig. 8. IGHV repertoire analysis of thymic B cell subsets in human neonates. A series of ecological indexes were applied to the IGHV repertoire diversity to show how evenly receptor sequences (rearrangements) are distributed amongst the three different groups of thymic B cells. Values near to 0 represent a completely even sequence, while values near to 1 represent a monoclonal sample. a Simpson clonality index in CD21+CD35+, CD21-CD35- and CD21-CD35-CD138+ thymic B cell subsets (n=5). **b** Simpson's D index in CD21+CD35+, CD21-CD35- and CD21-CD35-CD138+ thymic B cell subsets (n=5). **c** iChaos1 in CD21 +CD35+, CD21-CD35- and CD21-CD35-CD138+ thymic B cell subsets (n=5). **d** Efron Thisted Estimator in CD21 +CD35+, CD21-CD35- and CD21-CD35-CD138+ thymic B cell subsets (n=5). **e** VH gene usage (%) of CD21+CD35 +, CD21-CD35- and CD21-CD35-CD138+ thymic B cell subsets ordered by age (n=5). **f** VH gene usage (%) of CD21+CD35+, CD21-CD35- and CD21-CD35-CD138+ thymic B cell subsets ordered by group (n=5).

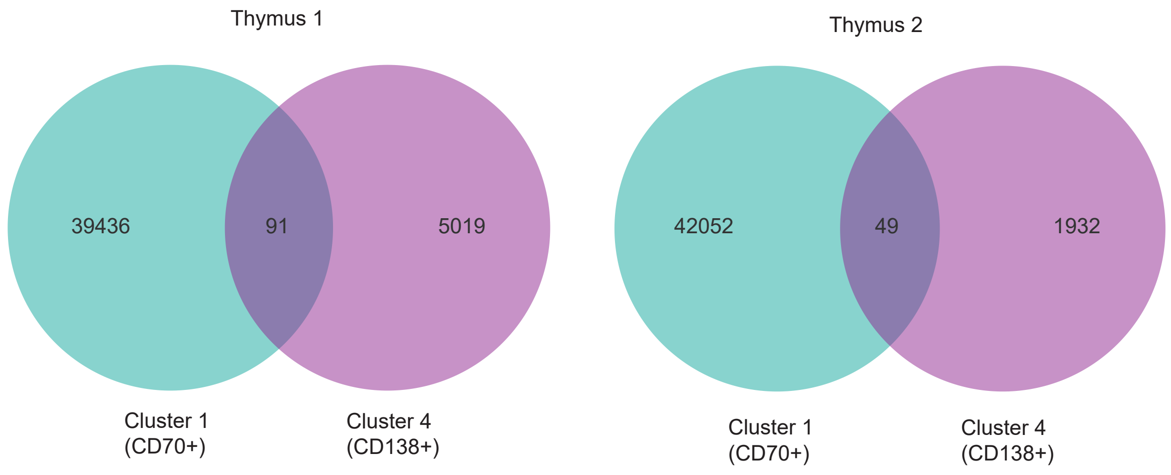

Supplementary Fig. 9. IGHV repertoire analysis of Cluster 1 and Cluster 4 in the thymus of human neonates. Venn Diagram showing shared nucleotide IGHV sequences between Cluster 1 (CD19+CD70+CD138-) and Cluster 4 (CD19+CD138+) in two different neonatal thymus specimens. Examples of shared sequences can be found in Supplementary Table 4.

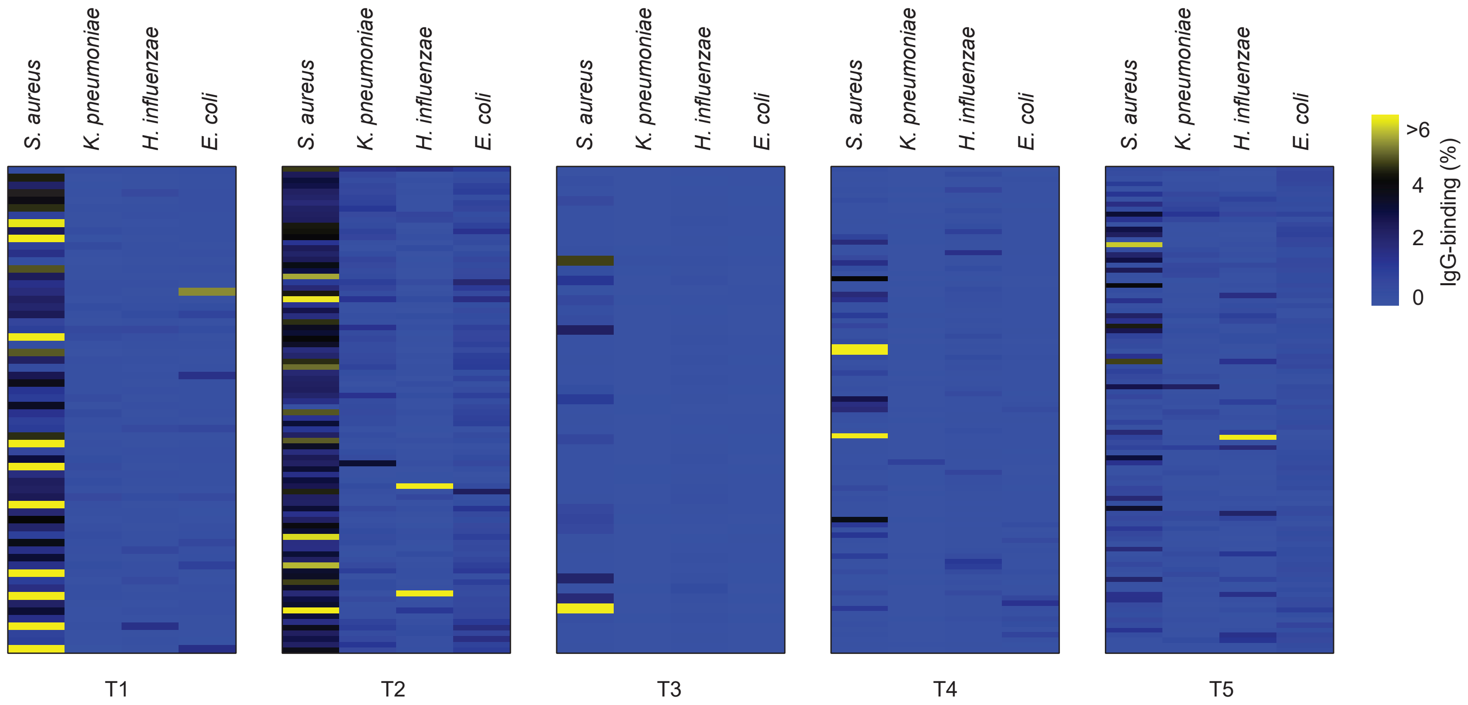

Supplementary Fig. 10. High throughput screening of recombinant monoclonal antibodies (rAbs) generated from thymic plasma cells in human neonates. Heat map representation of reactivity of recombinant monoclonal antibodies generated from thymic plasma cells to *Staphylococcus aureus*, *Klebsiella pneumoniae*, *Haemophilus influenzae* and *Escherichia coli*. Results are expressed as percentage of antibody-binding.

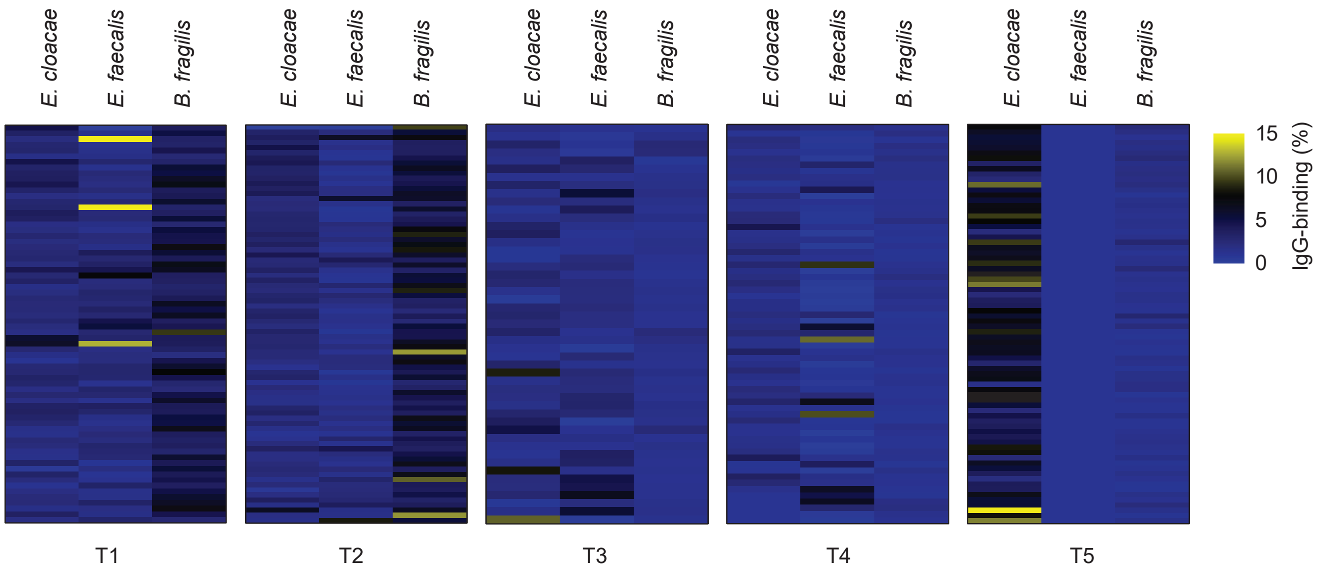

Supplementary Fig. 11. High throughput screening of recombinant monoclonal antibodies (rAbs) generated from thymic plasma cells in human neonates. Heat map representation of reactivity of recombinant monoclonal antibodies generated from thymic plasma cells to *Enterobacter cloacae*, *Enterococcus faecalis*, and *Bacteroides fragilis*. Results are expressed as percentage of antibody-binding.

Supplementary Table 1. B1 and B2 Signature. Two-sided Wald-test with Benjamini-Hochberg false discovery ration (FDR) adjustment (n=5).

Thymic CD21-CD35- vs Cord Blood CD19+			
**B1 signature**	**log2 FoldChange**	**padj**	**Outcome**
ZBTB32	6.70	1.55E-42	UP
BHLHE41	5.56	4.03E-23	UP
PLSCR1	4.25	4.21E-41	UP
GPR55	3.56	2.07E-20	UP
MYO1D	3.50	3.70E-26	UP
PSTPIP2	1.48	0.02	UP
TBC1D9	-2.85	1.11E-19	DOWN
LYSMD2	< 1	> 0.05	No difference
AHR	< 1	> 0.05	No difference
CSF2RB	< 1	> 0.05	No difference
CD300LF	NA	NA	No detected
FAM160A1	NA	NA	No detected
CYP11A1	NA	NA	No detected
FOLR4	NA	NA	No detected

Thymic CD21+CD35+ vs Cord Blood CD19+			
**B1 signature**	**log2 FoldChange**	**padj**	**Outcome**
ZBTB32	4.00	9.4E-09	UP
BHLHE41	4.15	4.57E-10	UP
PLSCR1	3.93	1.3E-38	UP
GPR55	2.03	6.96E-04	UP
MYO1D	2.03	2.91E-05	UP
PSTPIP2	< 1	> 0.05	No differences
TBC1D9	-1.52	4.33E-13	DOWN
LYSMD2	2.02	5.88E-15	UP
AHR	< 1	> 0.05	No difference
CSF2RB	< 1	> 0.05	No difference
CD300LF	NA	NA	No detected
FAM160A1	NA	NA	No detected
CYP11A1	NA	NA	No detected
FOLR4	NA	NA	No detected

Thymic CD21-CD35- vs Cord Blood CD19+			
**B2 signature**	**log2 FoldChange**	**padj**	**Outcome**
CSRP2	-2.08	0.03	DOWN
GDF11	< 1	> 0.05	No difference
SOX4	< 1	> 0.05	No difference
CPM	< 1	> 0.05	No difference
CCR6	< 1	> 0.05	No difference
LRRK2	< 1	> 0.05	No difference
DENND3	< 1	> 0.05	No difference
FAM101B	NA	NA	No detected
FCER2A	NA	NA	No detected
ICOSL	NA	NA	No detected
ZFP608	NA	NA	No detected
1300014i06rik	NA	NA	No detected
NEURL3	NA	NA	No detected
EMID1	NA	NA	No detected

Thymic CD21+CD35+ vs Cord Blood CD19+			
**B2 signature**	**log2 FoldChange**	**padj**	**Outcome**
CSRP2	< 1	> 0.05	No difference
GDF11	< 1	> 0.05	No difference
SOX4	< 1	> 0.05	No difference
CPM	< 1	> 0.05	No difference
CCR6	1.02	0.00	UP
LRRK2	< 1	> 0.05	No difference
DENND3	< 1	> 0.05	No difference
FAM101B	NA	NA	No detected
FCER2A	NA	NA	No detected
ICOSL	NA	NA	No detected
ZFP608	NA	NA	No detected
1300014i06rik	NA	NA	No detected
NEURL3	NA	NA	No detected
EMID1	NA	NA	No detected

Supplementary Table 2. GSEA Upregulated pathways in CD21-CD35- vs CD21+CD35+ thymic B cells.

NAME	SIZE	ES	NES	NOM p-value*	FDR q-value	FWER p-value
GO_CELL_CYCLE	218	0.27	4.39	0	0	0
GO_PHAGOCYTOSIS_RECOGNITION	15	0.78	3.55	0	0	0
GO_POSITIVE_REGULATION_OF_B_CELL_ACTI	24	0.58	3.35	0	0	0
GO_DEFENSE_RESPONSE	156	0.21	2.94	0	0	0
GO_HUMORAL_IMMUNE_RESPONSE_MEDIATE	21	0.53	2.94	0	0	0
GO_REGULATION_OF_B_CELL_ACTIVATION	37	0.40	2.83	0	0	0
GO_B_CELL_RECEPTOR_SIGNALING_PATHWA	24	0.49	2.82	0	0	0
GO_DEFENSE_RESPONSE_TO_OTHER_ORGAN	47	0.34	2.77	0	0	0
GO_B_CELL_MEDIATED_IMMUNITY	29	0.42	2.74	0	0	0
GO_DEFENSE_RESPONSE_TO_BACTERIUM	35	0.40	2.74	0	0	0
GO_REGULATION_OF_LYMPHOCYTE_ACTIVATI	87	0.25	2.65	0	1.14E-04	0.001
GO_INNATE_IMMUNE_RESPONSE	91	0.24	2.61	0	1.04E-04	0.001
GO_HUMORAL_IMMUNE_RESPONSE	34	0.38	2.56	0	9.62E-05	0.001
GO_B_CELL_ACTIVATION	49	0.30	2.51	0	8.93E-05	0.001
GO_ADAPTIVE_IMMUNE_RESPONSE	76	0.24	2.36	0.002057613	7.38E-04	0.007
GO_RESPONSE_TO_ENDOPLASMIC_RETICULU	24	0.37	2.16	0	0.003369276	0.03
GO_RESPONSE_TO_BACTERIUM	79	0.21	2.15	0.002040816	0.003346522	0.032
GO_PHAGOCYTOSIS	44	0.26	2.08	0	0.004845634	0.05
GO_CELLULAR_RESPONSE_TO_LIPID	74	0.18	1.77	0.02053388	0.024468707	0.245
GO_REGULATION_OF_T_CELL_ACTIVATION	59	0.19	1.74	0.018595042	0.027708095	0.283

*Two-sided Kolmogorov–Smirnov test.

Supplementary Table 3. Number of productive templates in the BCR study.

Sample ID	Productive templates	Productive Rearrangements
T1-CD19+CD21-CD35-CD138+	99	76
T2-CD19+CD21-CD35-CD138+	532	420
T3-CD19+CD21-CD35-CD138+	1905	1498
T4-CD19+CD21-CD35-CD138+	1476	1159
T5-CD19+CD21-CD35-CD138+	521	405
T1-CD19+CD21-CD35-	4421	3748
T2-CD19+CD21-CD35-	3543	2718
T3-CD19+CD21-CD35-	10762	9030
T4-CD19+CD21-CD35-	5717	4520
T5-CD19+CD21-CD35-	40171	37664
T1-CD19+CD21+CD35+	6327	5408
T2-CD19+CD21+CD35+	7494	6156
T3-CD19+CD21+CD35+	25471	23140
T4-CD19+CD21+CD35+	16348	13871
T5-CD19+CD21+CD35+	46384	44175

Supplementary Table 4. Examples of shared nucleotide sequences between CD138+ and CD70+ subsets in the thymus.

**Thymus 1 (T72)**	Unique nucleotide shared sequence	Translated CDR3 sequence
Sequence 1	CAGAGATGATTCAAAGAACACGGCGTATCTGCAAATGAACAGCCTGAAAACCGAGGACACGGCCGTGTATTACTGTGCTAGACTTAGCAGCAGCTCCACCTATGATGCTTTTGATATCTGGGGCCAAGGG	ARLSSSSTYDAFDI
Sequence 2	CGACAAGTCCATCAGCACCGCCTACCTGCAGTGGAGCAGCCTGAAGGCCTCGGACACCGCCATGTATTACTGTGGGAGAGGCCATAGCAGCTCGTCCGCACATGATGGTTTTGATATCTGGGGCCAAGGG	GRGHSSSSAHDGFDI
Sequence 3	ATTCACCATCTCCAGAGACAACACCAAGAACTCACTATATCTGCAAATGAACAGCCTGAGAGTCGAGGACACGGCTGTGTATTACTGTGTGAGAGATCAATGGTGGGCTTTTGATGTCTGGGGCCAAGGG	VRDQWWAFDV
**Thymus 2 (T73)**		
Sequence 1	GGTCACCATGACCAGGGACACGTCCATCAGCACAGCCTACATGGAGCTGAGCAGGCTGAGATCTGACGACACGGCCGTGTATTACTGTGCGAAAGCAAGGGGGGATGCTTTTGATATCTGGGGCCAAGGG	AKARGDAFDI
Sequence 2	CATCAGCACCGCCTACCTGCAGTGGAGCAGCCTGAAGGCCTCGGACACCGCCATGTATTACTGTGCGAGAAAGCTCTTTGGTTCGGGGAGTTATTACTACTACTACGGTATGGACGTCTGGGGCCAAGGG	ARKLFGSGSYYYYYGMDV
Sequence 3	CAGAGACAACGCCAAGAACTCACTGTATCTGCAAATGAACAGCCTGAGAGCCGAGGACACGGCCGTGTATTACTGTGCGAAAGATCGGGGTATAGTGGCTACGTCCCCATGGGACTACTGGGGCCAGGGA	AKDRGIVATSPWDY
**Thymus 3 (T135)**		
Sequence 1	GGACACGTCCACGAGCACAGTCTACATGGAGCTGAGCAGCCTGAGATCTGAGGACACGGCCGTGTATTACTGTGCGAGAGATAGGGGCTCTTATGGAGCAATCTATGCTTTTGATATCTGGGGCCAAGGG	ARDRGSYGAIYAFDI
Sequence 2	CCAGTTCTCCCTGAAGCTGAGCTCTGTGACTGCCGCGGACACGGCCGTGTATTACTGTGCCAGGACATCGAATATGGTTCGGGGAGTTATTATAACGAGTGGGGACTGGTTCGACCCCTGGGGCCAGGGA	ARTSNMVRGVIITSGDWFDP
Sequence 3	CACCATCTCCAGGGACAACGCCAAGAACTCACTGTATCTGCAAATGAACAGCCTGAGAGCCGAGGACACGGCCGTGTATTACTGTGCGAGTGGATGGTTCGGGGAGTTATTTGACTACTGGGGCCAGGGA	ASGWFGELFDY

Supplementary Table 5. List of antibodies used in the study with each dilution.

Antibody	Dilution
Anti- CD3 BV570 , Supplier Biolegend , Clone UCHT1 , Cat # 300435 ;	1:50
Anti- CD3 BV786 , Supplier BD Biosciences , Clone SK7 , Cat # 563800 ;	1:50
Anti- CD45 Qdot800 , Supplier Thermo Fisher Scientific , Clone HI30 , Cat # Q10156 ;	1:50
Anti- CD19 PECy7 , Supplier Tonbo Biosciences , Clone HIB19 , Cat # 20-0199 ;	1:50
Anti- CD21 BV711 , Supplier BD Biosciences , Clone B-ly4 , Cat # 563163 ;	1:50
Anti- CD21 PECy5 , Supplier BD Biosciences , Clone B-ly4 , Cat # 551064 ;	1:50
Anti- CD21 V450 , Supplier BD Biosciences , Clone B-ly4 , Cat # 561381 ;	1:50
Anti- CD35 PE , Supplier BD Biosciences , Clone E11 , Cat # 559872 ;	1:50
Anti- CD35 FITC , Supplier BD Biosciences , Clone E11 , Cat # 555452 ;	1:40
Anti- CD38 PerCP , Supplier Biolegend , Clone HIT2 , Cat # 303519 ;	1:50
Anti- CD38 BV650 , Supplier BD Biosciences , Clone HIT2 , Cat # 740574 ;	1:50
Anti- CD138 VB515 , Supplier Miltenyi Biotec , Clone 44F9 , Cat # 130-119-933 ;	1:50
Anti- CD138 PE , Supplier Miltenyi Biotec , Clone 44F9 , Cat # 130-119-840 ;	1:50
Anti- CD70 APC, Supplier Biolegend , Clone 113-16 , Cat # 355109 ;	1:50
Anti- CD27 APCCy7 , Supplier Tonbo Biosciences , Clone O323 , Cat # 25-0279-T100 ;	1:50
Anti- IgG AF700 , Supplier BD Biosciences , Clone G8-145 , Cat # 561298 ;	1:50
Anti- IgA APC , Supplier Miltenyi Biotec , Clone IS11-8E11 , Cat # 130-113-472 ;	1/100
Anti- IgM BV421 , Supplier BD Biosciences , Clone MHM-88 , Cat # 314516 ;	1:50
Anti- IgD BV510 , Supplier BD Biosciences , Clone IA6-2 , Cat # 563034 ;	1:50
Anti- IgE BV480 , Supplier BD Biosciences , Clone G7-26 , Cat # 746540 ;	1:50
Anti- CD80 BV711 , Supplier Biolegend , Clone 2D10 , Cat # 305235 ;	1:50
Anti- CD86 AF647 , Supplier Biolegend , Clone IT2.2 , Cat # 305415 ;	1:50
Anti- PD1 PE-Dazzle594 , Supplier Biolegend , Clone EH12.2H7 , Cat # 329939 ;	1:50
Anti- CD39 BV650 , Supplier BD Biosciences , Clone TU66 , Cat # 563681 ;	1:50
Anti- CD59 PE , Supplier Biolegend , Clone H19 , Cat # 304707 ;	1:50
Anti- CD269 PerCPCy5.5 , Supplier Biolegend , Clone 19F2 , Cat # 357509 ;	1:50
Anti- XBP1S PE , Supplier BD Biosciences , Clone Q3-695 , Cat # 562642 ;	1:50
Anti- IRF4 PerCPCy5.5 , Supplier Biolegend , Clone IRF4.3E4 , Cat # 646415 ;	1:50
Anti- BLIMP1 AF647 , Supplier BD Biosciences , Clone 6D3 , Cat # 565274 ;	1:50
Anti- KI67 FITC , Supplier Thermo Fisher Scientific , Clone SolA15 , Cat # 11-5698-82 ;	1/100
Anti- CD69 PE-Cy5 , Supplier Biolegend , Clone FN50 , Cat # 310907 ;	1:50
Anti-human IgG FITC, Supplier Fisher Thermo Scientific, Polyclonal, Cat # A24477 ;	1/200
Anti-human CD19, Supplier Leica Biosystems, Clone BT51E, Cat # NCL-L-CD19-163 ;	1/200
Anti-human CD31, Supplier Abcam, Clone C31.3 + JC/70A, Cat # ab199012 ;	1/100
Anti-human cytokeratin, Supplier Abcam, Clone PCK-26, Cat # ab6401 ;	1/200
Anti-human CD138, Supplier Leica Biosystems, Clone MI15, Cat # PA0088 ;	Ready to use
Opal 7-Color IHC Kit, Supplier Akoya Biosciences, Cat # NEL801001KT ;	1/100

Supplementary Table 6. List of primers used to generate recombinant antibodies.

	Name	Sequence
1	HIGH Screen F	GGGCTGGAGCTCTGGCTC
2	HIGH Screen R2	CCAGGGGGAAGACCGATGGG
3	Hkappa Screen F	CCTTGCTCTGGATCTCTGGTGC
4	Hkappa Screen R	GTGCTGTCCTTGCTGTCCTGC
5	Hlambda Screen F	GGGTCCTGGGCCCAGTCTGTG
6	Hlambda Screen R	CACCAGTGTGGCCTTGTTGGCTT
