# Supplementary material for: Intrathymic differentiation of natural antibody-producing plasma cells in human neonates
Source: Nat Commun. 2021 Oct 1;12:5761. doi: 10.1038/s41467-021-26069-2 (PMC8486820; doi:10.1038/s41467-021-26069-2)
Supplement: Supplementary file 2 — Description of Additional Supplementary Files [file 41467_2021_26069_MOESM2_ESM.pdf]

Description of Additional Supplementary Files

File Name: Supplementary Data 1
Description: **(a)** Transcriptome dataset of cord blood CD19+ vs thymic CD21-CD35-. Two-sided Wald-test with Benjamini-Hochberg false discovery ration (FDR) adjustment (n=5). **(b)** Transcriptome dataset of cord blood CD19+ vs thymic CD21+CD35+. Two-sided Wald-test with Benjamini-Hochberg false discovery ration (FDR) adjustment (n=5).

File Name: Supplementary Data 2
Description: Transcriptome dataset of thymic CD21+CD35+ vs thymic CD21-CD35-. Two-sided Wald-test with Benjamini-Hochberg false discovery ration (FDR) adjustment (n=5).

File Name: Supplementary Data 3
Description: List of DEG by cluster. Two-sided MAST (Model-based Analysis of Single-cell Transcriptomics) test with Bonferroni correction.
