# Supplementary material for: Intrathymic differentiation of natural antibody-producing plasma cells in human neonates
Source: Nat Commun. 2021 Oct 1;12:5761. doi: 10.1038/s41467-021-26069-2 (PMC8486820; doi:10.1038/s41467-021-26069-2)
Supplement: Supplementary file 6 — Reporting Summary [file 41467_2021_26069_MOESM6_ESM.pdf]

nature research

Corresponding author(s): Emmanuel Zorn

Last updated by author(s): Jun 28, 2021

# Reporting Summary

Nature Research wishes to improve the reproducibility of the work that we publish. This form provides structure for consistency and transparency in reporting. For further information on Nature Research policies, see our Editorial Policies and the Editorial Policy Checklist.

## Statistics

For all statistical analyses, confirm that the following items are present in the figure legend, table legend, main text, or Methods section.

| n/a | Confirmed | |
|---|---|---|
| ☐ | ☒ | The exact sample size ($n$) for each experimental group/condition, given as a discrete number and unit of measurement |
| ☐ | ☒ | A statement on whether measurements were taken from distinct samples or whether the same sample was measured repeatedly |
| ☐ | ☒ | The statistical test(s) used AND whether they are one- or two-sided<br>*Only common tests should be described solely by name; describe more complex techniques in the Methods section.* |
| ☒ | ☐ | A description of all covariates tested |
| ☒ | ☐ | A description of any assumptions or corrections, such as tests of normality and adjustment for multiple comparisons |
| ☐ | ☒ | A full description of the statistical parameters including central tendency (e.g. means) or other basic estimates (e.g. regression coefficient) AND variation (e.g. standard deviation) or associated estimates of uncertainty (e.g. confidence intervals) |
| ☐ | ☒ | For null hypothesis testing, the test statistic (e.g. $F$, $t$, $r$) with confidence intervals, effect sizes, degrees of freedom and $P$ value noted<br>*Give P values as exact values whenever suitable.* |
| ☒ | ☐ | For Bayesian analysis, information on the choice of priors and Markov chain Monte Carlo settings |
| ☒ | ☐ | For hierarchical and complex designs, identification of the appropriate level for tests and full reporting of outcomes |
| ☒ | ☐ | Estimates of effect sizes (e.g. Cohen's $d$, Pearson's $r$), indicating how they were calculated |

*Our web collection on statistics for biologists contains articles on many of the points above.*

## Software and code

Policy information about availability of computer code

| | |
|---|---|
| Data collection | EazyReader software version 18.9.<br>10X Genomics Cell Ranger v2.1.1.<br>Gatan Microscopy Suite Software version 2.<br>Vectra version 3.0.<br><br>Custom codes are publicly available at https://github.com/Dr-Cordero/natcomms-2021 |
| Data analysis | RStudio version 3.6.0.<br>sva package version 3.40.0.<br>DESEeq2 package 1.30.0.<br>ggplot2 package 3.3.3.<br>Seurat package version 3.0.<br>GSEA software version 4.0.1.<br>Velocyto package version 0.17.<br>MaxQuant software package v2.0.1.0.<br>Perseus software v1.6.15.0.<br>FCS Express Research Edition version 6.0.<br>ImageJ software version 1.52a.<br>InForm cell analysis software version 2.4.6.<br>Microsoft Excel version 16.0.<br>GraphPad Prism software version 7.0. |

For manuscripts utilizing custom algorithms or software that are central to the research but not yet described in published literature, software must be made available to editors and reviewers. We strongly encourage code deposition in a community repository (e.g. GitHub). See the Nature Research guidelines for submitting code & software for further information.

## Data

Policy information about availability of data

All manuscripts must include a data availability statement. This statement should provide the following information, where applicable:

- Accession codes, unique identifiers, or web links for publicly available datasets
- A list of figures that have associated raw data
- A description of any restrictions on data availability

The processed data and transcriptome datasets for both RNA-Seq and Single-Cell RNA-Seq generated during this study are available on NCBI GEO with the accession number: GSE152453 (https://www.ncbi.nlm.nih.gov/geo/query/acc.cgi?acc=GSE152453) and GSE153117 (https://www.ncbi.nlm.nih.gov/geo/query/acc.cgi?acc=GSE153117), respectively. The database for the GRCh38 human reference genome is available at useast.ensembl.org/Homo_sapiens/Info/Index. DNA sequencing data used for BCR repertoire analysis is available at Adaptive Biotechnology ImmunoACCESS doi: 10.21417/HC2021NC. There is no restriction on data availability.

# Field-specific reporting

Please select the one below that is the best fit for your research. If you are not sure, read the appropriate sections before making your selection.

☒ Life sciences ☐ Behavioural & social sciences ☐ Ecological, evolutionary & environmental sciences

For a reference copy of the document with all sections, see nature.com/documents/nr-reporting-summary-flat.pdf

# Life sciences study design

All studies must disclose on these points even when the disclosure is negative.

| | |
|---|---|
| Sample size | The sample size was determined by the initial longitudinal flow cytometry experiment using twenty-one human specimens, where five representative samples distributed during the first months were chosen to ensure a robust statistical analysis. For downstream applications such as sequencing, we chose the same five samples per group and selected tissue, which were demonstrated to be representative of neonatal thymuses and suitable sample size. For single cell experiments, three replicates from each age group was added obtaining no significant differences. |
| Data exclusions | No data were excluded during the analysis. |
| Replication | All the experiments were performed in triplicate to ensure reproducibility. All attempts at replication were successful. |
| Randomization | Samples were allocated in different groups depending on age and tissue source. |
| Blinding | The investigators were blinded to group allocation during the data collection and analysis. |

# Reporting for specific materials, systems and methods

We require information from authors about some types of materials, experimental systems and methods used in many studies. Here, indicate whether each material, system or method listed is relevant to your study. If you are not sure if a list item applies to your research, read the appropriate section before selecting a response.

## Materials & experimental systems

| n/a | Involved in the study | |
|---|---|---|
| ☐ | ☒ | Antibodies |
| ☐ | ☒ | Eukaryotic cell lines |
| ☒ | ☐ | Palaeontology and archaeology |
| ☒ | ☐ | Animals and other organisms |
| ☒ | ☐ | Human research participants |
| ☒ | ☐ | Clinical data |
| ☒ | ☐ | Dual use research of concern |

## Methods

| n/a | Involved in the study | |
|---|---|---|
| ☒ | ☐ | ChIP-seq |
| ☐ | ☒ | Flow cytometry |
| ☒ | ☐ | MRI-based neuroimaging |

## Antibodies

Antibodies used

Anti- CD3 BV570 , Supplier Biolegend , Clone UCHT1 , Cat # 300435 ;
Anti- CD3 BV786 , Supplier BD Biosciences , Clone SK7 , Cat # 563800 ;
Anti- CD45 Qdot800 , Supplier Thermo Fisher Scientific , Clone HI30 , Cat # Q10156 ;
Anti- CD19 PECy7 , Supplier Tonbo Biosciences , Clone HIB19 , Cat # 20-0199 ;
Anti- CD21 BV711 , Supplier BD Biosciences , Clone B-ly4 , Cat # 563163 ;
Anti- CD21 PECy5 , Supplier BD Biosciences , Clone B-ly4 , Cat # 551064 ;

Anti- CD21 V450 , Supplier BD Biosciences , Clone B-ly4 , Cat # 561381 ;
Anti- CD35 PE , Supplier BD Biosciences , Clone E11 , Cat # 559872 ;
Anti- CD35 FITC , Supplier BD Biosciences , Clone E11 , Cat # 555452 ;
Anti- CD38 PerCP , Supplier Biolegend , Clone HIT2 , Cat # 303519 ;
Anti- CD38 BV650 , Supplier BD Biosciences , Clone HIT2 , Cat # 740574 ;
Anti- CD138 VB515 , Supplier Miltenyi Biotec , Clone 44F9 , Cat # 130-119-933 ;
Anti- CD138 PE , Supplier Miltenyi Biotec , Clone 44F9 , Cat # 130-119-840 ;
Anti- CD70 APC, Supplier Biolegend , Clone 113-16 , Cat # 355109 ;
Anti- CD27 APCCy7 , Supplier Tonbo Biosciences , Clone O323 , Cat # 25-0279-T100 ;
Anti- IgG AF700 , Supplier BD Biosciences , Clone G8-145 , Cat # 561298 ;
Anti- IgA APC , Supplier Miltenyi Biotec , Clone IS11-8E11 , Cat # 130-113-472 ;
Anti- IgM BV421 , Supplier BD Biosciences , Clone MHM-88 , Cat # 314516 ;
Anti- IgD BV510 , Supplier BD Biosciences , Clone IA6-2 , Cat # 563034 ;
Anti- IgE BV480 , Supplier BD Biosciences , Clone G7-26 , Cat # 746540 ;
Anti- CD80 BV711 , Supplier Biolegend , Clone 2D10 , Cat # 305235 ;
Anti- CD86 AF647 , Supplier Biolegend , Clone IT2.2 , Cat # 305415 ;
Anti- PD1 PE-Dazzle594 , Supplier Biolegend , Clone EH12.2H7 , Cat # 329939 ;
Anti- CD39 BV650 , Supplier BD Biosciences , Clone TU66 , Cat # 563681 ;
Anti- CD59 PE , Supplier Biolegend , Clone H19 , Cat # 304707 ;
Anti- CD269 PerCPCy5.5 , Supplier Biolegend , Clone 19F2 , Cat # 357509 ;
Anti- XBP1S PE , Supplier BD Biosciences , Clone Q3-695 , Cat # 562642 ;
Anti- IRF4 PerCPCy5.5 , Supplier Biolegend , Clone IRF4.3E4 , Cat # 646415 ;
Anti- BLIMP1 AF647 , Supplier BD Biosciences , Clone 6D3 , Cat # 565274 ;
Anti- KI67 FITC , Supplier Thermo Fisher Scientific , Clone SolA15 , Cat # 11-5698-82 ;
Anti- CD69 PE-Cy5 , Supplier Biolegend , Clone FN50 , Cat # 310907 ;

Anti-human IgG, Supplier Mabtech, Clone MT91/145, Cat # 3850-3-250;
Anti-human IgM, Supplier Mabtech, Clone MT11/12, Cat # 3880-3-250 ;
Anti-human IgA, Supplier Mabtech, Clone MT57, Cat # 3860-3-250 ;
Anti-human IgE, Supplier Mabtech, Clone 107, Cat # 3810-3-250 ;

Biotinylated anti-human IgG, Supplier Mabtech, Clone MT78/145, Cat # 3850-6-250 ;
Biotinylated anti-human IgM, Supplier Mabtech, Clone MT22, Cat # 3880-6-250 ;
Biotinylated anti-human IgA, Supplier Mabtech, Clone MT20, Cat # 3860-6-250 ;
Biotinylated anti-human IgE, Supplier Mabtech, Clone 107/182/101, Cat # 3810-8-250 ;

Anti-human IgG FITC, Supplier Fisher Thermo Scientific, Polyclonal, Cat # A24477 ;

Anti-human CD19, Supplier Leica Biosystems, Clone BT51E, Cat # NCL-L-CD19-163 ;
Anti-human CD31, Supplier Abcam, Clone C31.3 + JC/70A, Cat # ab199012 ;
Anti-human cytokeratin, Supplier Abcam, Clone PCK-26, Cat # ab6401 ;
Anti-human CD138, Supplier Leica Biosystems, Clone MI15, Cat # PA0088 ;
Opal 7-Color IHC Kit, Supplier Akoya Biosciences, Cat # NEL801001KT ;

Validation

All the primary antibodies were validated for their specific application in humans by the manufacturer. This validation data is available on each manufacturer's website. Antibodies for multiplex immunofluorescence were validated individually before combining them.

# Eukaryotic cell lines

Policy information about cell lines

| | |
|---|---|
| Cell line source(s) | FreeStyle™ 293-F Cells was purchased from Thermo Fisher Scientific. |
| Authentication | This cell line was certified and authenticated using ATCC Short Tandem Repeat profiling by Thermo Fisher Scientific. |
| Mycoplasma contamination | This cell line was tested negative for mycoplasma contamination. |
| Commonly misidentified lines (See ICLAC register) | No commonly misidentified cell lines used in the study. |

# Flow Cytometry

## Plots

Confirm that:

☒ The axis labels state the marker and fluorochrome used (e.g. CD4-FITC).

☒ The axis scales are clearly visible. Include numbers along axes only for bottom left plot of group (a 'group' is an analysis of identical markers).

☒ All plots are contour plots with outliers or pseudocolor plots.

☒ A numerical value for number of cells or percentage (with statistics) is provided.

## Methodology

| | |
|---|---|
| Sample preparation | Thymic tissue was collected in cold phosphate-buffered saline (PBS) and washed extensively to remove the blood. The tissue was homogenized using a gentleMACS tissue dissociator (Miltenyi Biotec) and filtered through a 40-µm cell strainer (BD Biosciences). Peripheral blood mononuclear cells (PBMCs) were isolated from cord blood and adult blood by Ficoll density gradient using Ficoll-PaquePLUS (GE HealthCare). Thymocytes, cord blood and adult blood cells were suspended RPMI media containing 10% FCS and washed in PBS. B cells were isolated by magnetic cell sorting with EasySep Human B Cell Enrichment Kit (Stem Cell Technologies) following the manufacturer's instructions and stained in PBS with 2% FCS for 45min with the each fluorochrome conjugated antibodies, washed and acquired. For intracellular staining, cells were fixed and permeabilized using Transcription Factor Staining Buffer Set (eBioscience) following the manufacturer's instructions prior to staining. Cells were washed in cold PBS with 2% FCS, filtered through a 70µm cell strainer and acquired. |
| Instrument | Cytek Aurora and BD LSRFortessa |
| Software | FCS Express version 6.0 |
| Cell population abundance | Most of the B cells in blood and cord blood (up to 97%) was positive for CD21 and CD35. In the case of thymic B cells, between 10 and 40% of them were either CD21+CD35+ or CD21-CD35-. |
| Gating strategy | The initial cell suspension was gated on the FSC/SSC plot avoiding debris and dead cells, then gated cells were selected for singlets. After this second gate, we selected CD3-CD19+ as B cells. Later, this population of CD19+ cells was divided into CD21+CD35+ and CD21-CD35- for the downstream analysis. |

☒ Tick this box to confirm that a figure exemplifying the gating strategy is provided in the Supplementary Information.
